# Long-term intravital subcellular imaging with confocal scanning light-field microscopy

**DOI:** 10.1038/s41587-024-02249-5

**Published:** 2024-05-27

**Authors:** Zhi Lu, Siqing Zuo, Minghui Shi, Jiaqi Fan, Jingyu Xie, Guihua Xiao, Li Yu, Jiamin Wu, Qionghai Dai

**Affiliations:** 1 Department of Automation, Tsinghua University, Beijing, China; 2 Institute for Brain and Cognitive Sciences, Tsinghua University, Beijing, China; 3 Beijing Key Laboratory of Multi-dimension & Multi-scale Computational Photography (MMCP), Tsinghua University, Beijing, China; 4 IDG/McGovern Institute for Brain Research, Tsinghua University, Beijing, China; 5 Zhejiang Hehu Technology, Hangzhou, China; 6 Hangzhou Zhuoxi Institute of Brain and Intelligence, Hangzhou, China; 7 State Key Laboratory of Membrane Biology, Tsinghua University–Peking University Joint Center for Life Sciences, Beijing Frontier Research Center for Biological Structure, School of Life Sciences, Tsinghua University, Beijing, China; 8 Tsinghua Shenzhen International Graduate School, Tsinghua University, Shenzhen, China; 9 Shanghai AI Laboratory, Shanghai, China; 10 Beijing National Research Center for Information Science and Technology, Tsinghua University, Beijing, China

**Keywords:** Fluorescence imaging, Confocal microscopy

## Abstract

Long-term observation of subcellular dynamics in living organisms is limited by background fluorescence originating from tissue scattering or dense labeling. Existing confocal approaches face an inevitable tradeoff among parallelization, resolution and phototoxicity. Here we present confocal scanning light-field microscopy (csLFM), which integrates axially elongated line-confocal illumination with the rolling shutter in scanning light-field microscopy (sLFM). csLFM enables high-fidelity, high-speed, three-dimensional (3D) imaging at near-diffraction-limit resolution with both optical sectioning and low phototoxicity. By simultaneous 3D excitation and detection, the excitation intensity can be reduced below 1 mW mm^−^^2^, with 15-fold higher signal-to-background ratio over sLFM. We imaged subcellular dynamics over 25,000 timeframes in optically challenging environments in different species, such as migrasome delivery in mouse spleen, retractosome generation in mouse liver and 3D voltage imaging in *Drosophila*. Moreover, csLFM facilitates high-fidelity, large-scale neural recording with reduced crosstalk, leading to high orientation selectivity to visual stimuli, similar to two-photon microscopy, which aids understanding of neural coding mechanisms.

## Abstract (web summary)

A confocal microscopy technique enables high-resolution intravital imaging over multiple hours.

## Main

Intravital imaging^1–7^ is vital for studying diverse physiopathological processes, such as brain functions and immune responses, which necessitates high spatiotemporal resolution, high fidelity and low phototoxicity to capture fine structures and transient three-dimensional (3D) dynamics non-invasively^8–13^. An essential difference between in vitro and in vivo imaging is the presence of intense background fluorescence originating from out-of-focus signals and autofluorescence in scattered tissue or densely labeled samples, which poses a fundamental constraint on the imaging fidelity^14–16^. Confocal microscopy^17–19^ is the most widely used technique to address this problem with optical sectioning. By rejecting the out-of-focus fluorescence with a pinhole, slit or pinhole array, only the in-focus photons within a very shallow depth of field (DOF) are captured, leading to high signal-to-background ratio (SBR) in intravital imaging. However, these confocal approaches inevitably reduce system parallelization indicated by the effective data throughput per unit of time and result in strong phototoxicity due to repeated excitation of out-of-focus layers and intense laser illumination during 3D imaging. In contrast, light-field microscopy (LFM)^20–29^ maximizes the parallelization with low phototoxicity by exciting and imaging the entire volume within an extended DOF. By incorporating line-confocal illumination, confocal light-field microscopy (cLFM)^30^ not only can detect 3D photons in a parallel way but also suppress the background fluorescence out of the effective axial range. However, its Fourier LFM configuration fundamentally reduces the spatial resolution due to the loss of high-frequency information during pupil segmentation, restricting the application in observing subcellular dynamics. Recently, we proposed scanning LFM (sLFM)^31^ to increase the resolution up to the diffraction limit while maintaining the low phototoxicity by placing a drifting coded microlens array (MLA) at the image plane^31,32^. Although sLFM enables high-speed multi-site aberration correction with digital adaptive optics to maintain subcellular resolution in multi-cellular organisms, it still faces severe degradation with the existence of strong background fluorescence.

To address this problem, we propose confocal scanning LFM (csLFM) to achieve aberration-corrected high-speed 3D subcellular imaging with both optical sectioning and low phototoxicity by developing a line-confocal scheme upon our sLFM^31^. With direct synchronization of an axially elongated line-confocal illumination and the camera rolling shutter of multiple rows, csLFM can selectively collect fluorescent signals from in-focus volume within a compact system. Compared to sLFM, csLFM achieves 15-fold improvement in SBR for high-fidelity imaging of densely labeled samples while maintaining the same effective axial coverage, near-diffraction-limit resolution in complicated environments and two orders-of-magnitude reduction in photobleaching over spinning-disk confocal microscopy (SDCM). We constructed both inverted and upright csLFM systems to demonstrate their applications in diverse species, including zebrafish, *Drosophila* and mouse. Although large-scale 3D neural recording is one of the key applications of LFM, our results show that the crosstalk from background fluorescence severely degrades the fidelity of calcium responses at the single-cell level, leading to reduced orientation selectivity to visual stimuli. csLFM effectively removes the crosstalk and achieves similar performance to two-photon microscopy but with lower excitation power even below 1 mW mm^−^^2^ and much higher data throughput. Moreover, various subcellular dynamics are observed by csLFM in previously challenging environments, such as migrasome delivery in mouse spleen, retractosome generation in mouse liver and 3D voltage imaging at subcellular resolution in *Drosophila* with dense labeling, demonstrating its broad applications in the intravital study of large-scale intercellular interactions.

## Results

### Principle of csLFM

**Fig. 1:**
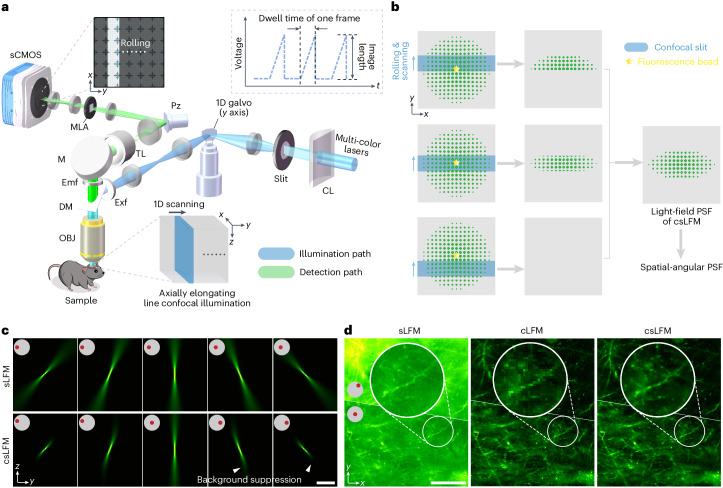
Schematic of csLFM. **a**, Diagram of csLFM system where an elongated line illumination is synchronized with the camera rolling shutter in the detection path. A cylindrical lens and an optical slit are used to converge the beam into an axially elongated line-confocal illumination with a galvo for lateral scanning. The piezoelectric tip/tilt platform and an MLA is used for scanning light-field imaging for the measurement of high-resolution spatial-angular components. CL, cylindrical lens; DM, dichroic mirror; Emf, emission filter; Exf, excitation filter; M, mirror; OBJ, objective; Pz, piezoelectric tip and tilt platform; sCMOS, scientific complementary metal-oxide semiconductor; TL, tube lens. **b**, Illustration of the confocal modulation on sLFM detection. The axially elongated line-confocal illumination is scanned to excite the fluorescence bead in the center of the FOV, with the rolling shutter synchronously reading out the active pixels, collecting emitted fluorescent photons from only in-focus regions. **c**, Comparisons of PSFs between sLFM and csLFM from several angles, with insets indicating corresponding sub-aperture. **d**, The raw measurements on a thick brain slice after pixel realignment for the comparison among sLFM, cLFM and csLFM. Two angles are visualized. Scale bars, 10 μm (**c**) and 50 μm (**d**).

To achieve both imaging parallelization and optical sectioning, we designed an elongated line-confocal illumination^33–35^ based on sLFM^31^. The camera rolling shutter is synchronized with the scanning illumination at high speed as the confocal slit to filter out background fluorescence without sacrificing the imaging speed in a compact system (Fig. 1a and Supplementary Fig. 1). To facilitate confocality, we set the slit size the same as the height of rolling shutter. The accurate synchronization contributes to a moving mask on light-field images followed by an integration process during exposure, which modulates the point spread functions (PSFs) at camera frame rate (Fig. 1b, Supplementary Fig. 2 and Methods). The MLA is placed at the imaging plane for light-field detection, along with a piezoelectric tip and tilt platform for high-speed periodic drifting to address the tradeoff between spatial resolution and angular resolution in LFM^31^ (Supplementary Video 1). After data acquisition, iterative tomography with digital adaptive optics^31^ same as previous sLFM can be applied for aberration-corrected 3D reconstruction with the modified confocal PSF. By keeping the photons focused within an extended DOF, csLFM provides an efficient way for 3D sensing with elongated PSFs along different angles (Fig. 1c). Therefore, the design of silt size entails a holistic assessment of the photon efficiency, axial coverage and background rejection performance. Only photons falling beyond the effective volume range need to be eliminated, which is different from traditional confocal microscopy requiring a small pinhole size for a shallow DOF. When we reduce the slit size together with the height of rolling shutter, csLFM will first maintain the same DOF and then gradually lose its effective axial coverage (Supplementary Fig. 3a–g). If we do not consider confocal illumination in PSF modeling, there will be artifacts at the out-of-focus planes during 3D reconstruction, especially for small slit sizes (Supplementary Fig. 3h,i). On the contrary, increasing the slit size will loosen the confocal constraint, degrading the capability of background rejection as indicated by the numerical simulations (Supplementary Fig. 4a–g). Similar optical sectioning can be obtained when the slit size is smaller than 11-times Airy units (AU) of the whole-objective numerical aperture (NA). Consequently, to maximize the 3D photon efficiency for low phototoxicity while maintaining the high SBR, we set the slit size as 11 AU for the angular resolution of 13 × 13 in csLFM, corresponding to the 13 × 13 sensor pixels after each microlens (Supplementary Fig. 4h,i). The axial coverage remains almost unchanged relative to sLFM with an effective DOF of 15 μm for 1.4-NA objective and similar optical sectioning capability compared to the silt size of 1 AU (Supplementary Figs. 3c–f and 4g).

By changing different background levels in numerical simulation, csLFM quantitatively achieved 12-dB improvement in SBR over sLFM (Supplementary Fig. 5). Although our previous study of computational background suppression^36^ can alleviate background contamination to some extent, the background fluorescence cannot be eliminated and will further reduce the axial resolution with the existence of strong background fluorescence (Supplementary Fig. 6). More importantly, the shot noise is positively correlated with the measured intensity, so it is difficult to be removed computationally. By physically rejecting the background fluorescence, csLFM can effectively increase the signal-to-noise ratio (SNR) to distinguish minute structures with subcellular resolution (Fig. 1d and Supplementary Fig. 7). One of the key advantages of sLFM is multi-site aberration correction with digital adaptive optics. Spatially non-uniform aberrations can be estimated based on the local disparities between different angular components and then corrected during 3D reconstruction with an accurate PSF model. However, strong background fluorescence will deteriorate the accuracy of wavefront estimation, leading to severe artifacts and reduced resolution (Supplementary Fig. 8). csLFM can address this problem because it effectively extracts signals from the background. Another challenge arises from the highly dynamic samples, leading to motion artifacts during physical scanning. Both time-weighted algorithm^31^ or optical-flow-based correction^32^ are also applicable to csLFM to eliminate the motion artifacts while maintaining high spatial resolution (Supplementary Fig. 9).

### Experimental characterization and analysis

**Fig. 2:**
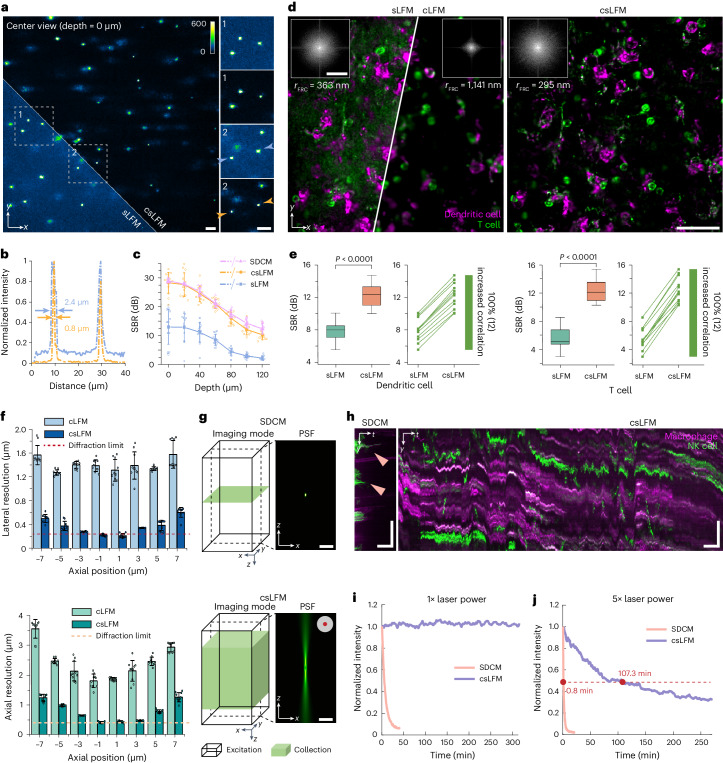
Experimental characterization of csLFM on SBR, resolution and phototoxicity. **a**, Center-view measurements and enlarged regions of mixture of 0.0005% 0.5-μm fluorescence beads, 1% intralipid and 1% agarose by sLFM and csLFM at the depth of 0 μm. **b**, Normalized intensity profiles along two beads. **c**, Curves of SBR versus different depths for sLFM, csLFM and SDCM. Data are represented as mean ± s.d. Twelve typical beads in each depth were involved. **d**, MIPs of dendritic cells and T cells in mouse spleens, acquired by sLFM, cLFM and csLFM. Insets show Fourier transforms with estimated resolutions by FRC. **e**, Box plot showing SBR comparisons between sLFM and csLFM. The box plot format: center line, median; box limits, lower and upper quartiles; and whiskers, 0th–100th percentiles excluding outliers. *P* values were calculated by two-sided paired *t*-test, *n* = 12 cells. *P* = 9.62 × 10^−13^ for dendritic cells and *P* = 2.77 × 10^−12^ for T cells. *P* < 0.05 was considered statistically significant. **f**, Box plots of the lateral and axial resolutions of cLFM and csLFM at different axial positions. *n* = 10 beads per depth. The resolution was estimated by measuring the FWHMs of intensity profiles across the beads. Dashed lines indicate diffraction limits at wavelength of 525 nm. Data are presented as mean values ± s.d. **g**, Illustration of imaging mode for SDCM and csLFM. Left, black borders and green boxes indicating the excitation range and the volume range to be collected. Right, the *x–z* slices of PSFs. **h**, Kymographs along *y–t* direction by SDCM for 40 min and csLFM for over 5 h in spleen imaging, with NK cells and macrophages labeled. Arrows indicate photobleaching. **i**, Curves of normalized average intensity versus time. **j**, Corresponding curves with five times more laser power. The measured photon count was similar for csLFM and SDCM. The excitation powers at the objective exit were set to the same (Supplementary Table 1). The dashed line indicates the value of 0.5. Scale bars, 10 μm (**a**), 40 μm, 3 μm^−1^ (**d**), 5 μm (**g**) and 30 μm spatially and 15 min temporally (**h**). MIP, maximum intensity projection.

For experimental validations, we compared csLFM with sLFM^31^ and cLFM^30^ on the same system (Methods) and state-of-the-art intravital microscopy, such as SDCM and two-photon microscopy. We implemented cLFM^30^ here with line-confocal illumination without two-dimensional (2D) periodic scanning. First, we evaluated the background rejection capability by imaging 500-nm-diameter fluorescence beads randomly distributed in a 3D tissue-mimicking phantom made of intralipid and agarose by different methods (Fig. 2a). Background fluorescence gradually overwhelmed the intensity of in-focus beads as the penetration depth increases, which degraded the imaging performance and limited the depth of sLFM (Supplementary Fig. 10a–c). Under different concentrations of intralipid, csLFM obtained an overall 12-dB improvement in terms of SBR with deeper penetration depth and about three-fold narrower lateral full width at half maximum (FWHM) profile along beads in raw measurements than sLFM (Fig. 2b). When compared to traditional slit confocal microscopy, csLFM captures four-dimensional (4D) spatial-angular measurements with an extended DOF along multiple angular PSFs. Therefore, the background fluorescence in the other orthogonal direction can still be distinguished based on the disparities between different angular views, achieving computational rejection of background fluorescence^36^. The statistical plots indicate that csLFM can achieve similar performance to SDCM (Andor Dragonfly 200) in terms of SBR (Fig. 2c and Supplementary Fig. 10d). Similar results can be obtained when we used an upright system to image vascular structures in the brain of an awake wild-type mouse injected with AF647 dye. By increasing the SBR, csLFM increased penetration depth from 140 μm to 280 μm in mouse cortex with more uniform resolution performance than sLFM (Supplementary Fig. 11).

Next, we imaged endogenous immune cells in a mouse spleen (Fig. 2d). On the one hand, there were many dense cells distributed at multiple layers of the spleen parenchyma with strong autofluorescence, causing background contamination in sLFM. On the other hand, cLFM achieved higher contrast but with low resolution. csLFM addressed both problems and distinguished subcellular structures, such as retraction fibers, with substantial SBR improvement (Fig. 2d,e). Then, we imaged a 300-μm-thick Thy1-YFP mouse brain slice (Supplementary Fig. 12a,b and part III of Supplementary Video 1). Even with dense dendrites and synapses intricately interlacing in 3D space, csLFM could distinctly resolve fine structures swamped in sLFM (Supplementary Fig. 12c,d). Because the background fluorescence is highly non-uniform, it is difficult to be removed by simple background subtraction (Supplementary Fig. 13). We further imaged the same thick brain slice to compare the imaging performance among csLFM, SDCM and two-photon microscopy. The objective NA was selected closely. Due to the resolution limit, two-photon microscopy exhibited about 1.8 times lower resolution than csLFM, and the axial resolution is over two times worse, making it unable to clearly distinguish minute structures. In contrast, csLFM can achieve nearly the same performance as SDCM. csLFM also obtained the imaging speed improvement of about 20 times than SDCM and two-photon microscopy, showing its higher data throughput through 3D parallel detection (Supplementary Fig. 14). In addition, we quantitatively characterized the resolution of csLFM by imaging 100-nm-diameter fluorescence beads. Our results show that csLFM reaches near-diffraction-limit resolution both laterally and axially within a 10-μm axial coverage (Fig. 2f and Supplementary Fig. 15). Compared to unscanned cLFM, csLFM enhanced the lateral resolution by over three times and the axial resolution by over two times. Such a high resolution is further verified by calculating the coefficients of Fourier ring correlation (FRC) based on the intravital images with about 295-nm lateral resolution for csLFM, which is even better than sLFM with the existence of strong background (Fig. 2d).

Although csLFM uses the rolling shutter to block out the out-of-focus photons, it still maintains high data parallelization within the effective DOF due to the elongated line-confocal illumination. By imaging the same fixed cell within the DOF under the same excitation power and frame dwell time, csLFM can capture similar numbers of photons compared to sLFM with a high photon efficiency of approximately 85% (Supplementary Fig. 16). In this case, csLFM can maintain the low-phototoxicity advantage of sLFM. On the contrary, the entire volume is excited in SDCM, but only the photons from a 2D layer with a small DOF can be collected by SDCM due to the presence of a pinhole or pinhole array to reject the out-of-focus photons (Fig. 2g). In contrast, the emitted photons from an extended DOF are effective in csLFM. If under the same excitation intensity, much more effective photons are collected by csLFM and sLFM than SDCM. To obtain similar photon outcome, more exposure time is required for SDCM compared to csLFM. For verification, we imaged immune cells labeled by flow antibodies in a living mouse spleen, which would be bleached quickly under intense excitation light in SDCM. Therefore, when imaging continuously at the same rate and average power intensity with similar photon counts for both methods (more exposure time required for SDCM; Supplementary Table 1), quick photobleaching appeared in the kymographs (*y–t* projections) of SDCM results, whereas csLFM exhibited no apparent bleaching for over 5 h (Fig. 2h,i). Even if the laser intensity was intentionally increased by a factor of 5, csLFM showed only slight photobleaching with the decay time 100-fold longer than that of SDCM (Fig. 2j).

Collectively, csLFM achieves high-fidelity high-speed 3D imaging with optical sectioning in complicated environments while maintaining subcellular resolution and low phototoxicity.

### Intercellular immune behaviors in living mouse spleens

Intravital imaging is critical to understand how the immune system works collaboratively in native states. A multitude of immune cells actively migrate and interact with each other. During the migration and interaction, immune cells leave long and thin retraction fibers and migrasomes to facilitate intercellular communication. These orchestrate dynamic and complicated cooperative behaviors to regulate immune responses^8,37^. The mammalian spleen is recognized as a prominent organ of the immune system, housing abundant resident or transient immune cells involved in executing immune reactions. However, the spleen remains a challenging environment for subcellular imaging due to its dense tissue, strong background fluorescence and sensitivity to photodamage^38,39^. csLFM exactly fills this niche.

**Fig. 3:**
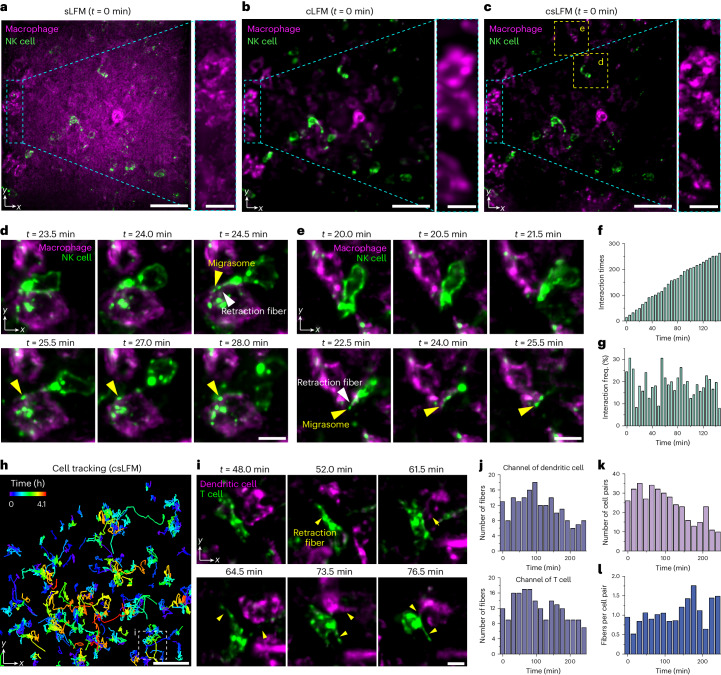
Subcellular interactions and migrasome delivery between multiple immune cells in mouse spleens. **a**–**c**, MIPs and enlarged regions of NK cells (green) and macrophages (magenta) in a living mouse spleen, acquired by sLFM (**a**), cLFM (**b**) and csLFM (**c**) at *t* = 0 min. **d**,**e**, Representative frames of csLFM showing that two independent NK cells migrated actively and produced migrasomes in neighboring macrophages. The arrows indicate retraction fibers and migrasomes formed on the tips of them. After the retraction fibers broke up, the contact-generated migrasomes were retained in macrophages. **f**, The accumulated interaction times between migrating NK cells and macrophages over time. **g**, The frequency of cell interactions over time. The frequency is defined as the ratio of the number of cells that engaged in interactions to the total cell number. **h**, Tracking traces of dendritic cells (magenta) and T cells (green) in a living mouse spleen captured by csLFM. The tracking map was obtained by Imaris 9.0.1, with the overall time length of 246 min. In total, 158 cells were tracked using temporal-coding trajectory, with the color bar indicating different timestamps. **i**, Representative magnified frames of csLFM showing the elaborate interactions of dendritic cell and T cell. Yellow arrows point to the elongating retraction fibers. **j**, Counts of retraction fibers generated by dendritic cells (upper row) and T cells (lower row) over time. The binning width is 15 min. The fibers were identified within the ROI shown in **h**. **k**, The number of cell pairs between dendritic cells and T cells that made contact over time. **l**, The average fiber number produced by each cell pair over time. For detailed data analysis in **f**, **g** and **j**–**l**, refer to the Methods section. Scale bars, 40 μm (original views in **a**–**c**,**h**) and 10 μm (enlarged views in **a**–**c**,**d**,**e**,**i**). MIP, maximum intensity projection.

We then compared csLFM to sLFM and cLFM by imaging immune cells in living mouse spleens. First, F4/80 and NK1.1 antibodies were intravenously injected into an anesthetized mouse to label macrophages and natural killer (NK) cells. Macrophages have a large size of over 20 µm and are broadly distributed in multiple compartments of the spleen^40^, leading to strong background during wide-field excitation (Fig. 3a). Although cLFM suppressed the background, the resolution would inevitably be reduced (Fig. 3b). On the contrary, csLFM resolved subcellular structures with considerable SBR improvement, which could be used to investigate migrasome functionality^41^ (Fig. 3c and part I of Supplementary Video 2). The delivery of migrasome in mammals was reported previously^31,42^, but it is confined to one type of immune cell (neutrophil). Whether migrasome could be a messenger between multiple immune cells is unknown. In this experiment, csLFM revealed that NK cells actively migrated in mouse spleen and sometimes interacted with macrophages through cellular protrusion extensions. When adhering to macrophages, NK cells generated retraction fibers and produced migrasomes inside the macrophages (Fig. 3d–g and part II of Supplementary Video 2). Similar cell-contacting processes through migrasomes were also validated by using a recently developed two-photon microscopy with a theoretical resolution of approximately 500 nm (ref. ^42^) but at a smaller data throughput fundamentally limited by the fluorescence decay time (Supplementary Fig. 17). Although the functions of immune-cell-derived migrasomes are still to be determined, we speculate that the delivery of migrasomes may be a collaborative mechanism of signaling, which probably strengthens the immune surveillance system.

Next, we further explored the intercellular communications between dendritic cells and T cells. With the low phototoxicity, csLFM sustainably monitored the spleen microenvironment over 4 h at high speed. Reduced background clearly unveiled cell shapes, whereby 158 highly motile immune cells could be continuously tracked (Fig. 3h). More interestingly, we found that a dendritic cell and a T cell were entangled and interacted. They showed very frequent contacts through elongating retraction fibers, as if talking and dancing with each other (Fig. 3i and Supplementary Video 3). Such discoveries reveal that different types of immune cells may communicate and work collaboratively through retraction fibers to enhance the innate adaptive immune systems. The retraction fibers produced by frequent interactions of dendritic cells and T cells were counted, suggesting that cell-to-cell contacts via fibers might be a common occurrence in the mammalian immune system with, on average, a single fiber per cell pair (Fig. 3j–l). The successful application of csLFM to spleen imaging provides an insightful way to better understand complex subcellular interactions and collaborative behaviors of endogenous immune cells and organelles and also demonstrates its potential in observing other mammalian organs.

### Retractosome biogenesis in mammals

**Fig. 4:**
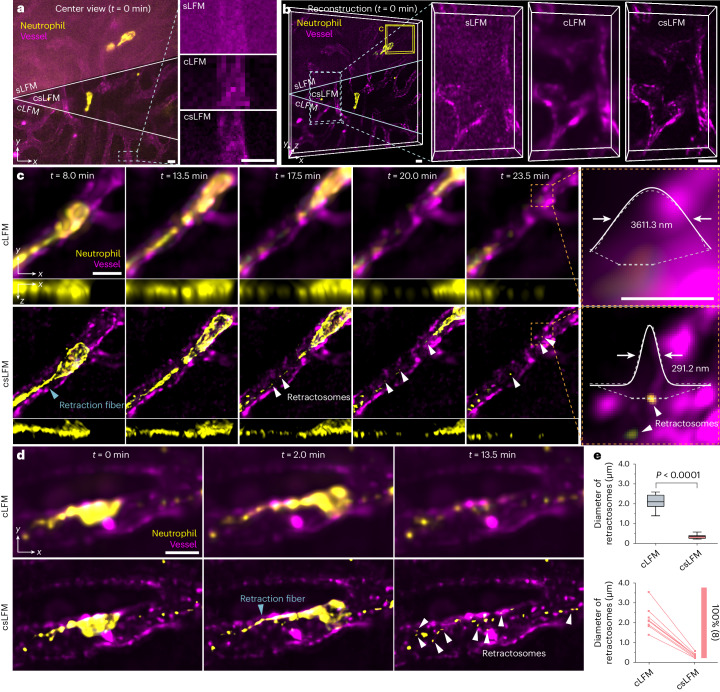
Long-term observation of retractosome formation in mouse livers. **a**,**b**, Raw center-view measurements without reconstruction (**a**) and 3D rendered volumes after reconstruction (**b**) of neutrophils and vessels in a living mouse liver, obtained by sLFM and csLFM at *t* = 0 min. The enlarged regions of vasculature are displayed on the right for detailed comparisons. **c**, Orthogonal MIPs of the yellow box in **b** by cLFM (upper) and csLFM (lower) at different timestamps, showing the process of retraction fiber breakage and retractosome formation behind a migrating neutrophil. Only neutrophil channel is displayed in *x*–*z* images. White arrows point to some retractosomes, and gray arrows point to retraction fibers. Further enlarged regions of retractosomes are shown in the right. Normalized intensity profiles along the marked line before and after Gaussian fit are plotted with measured FWHM value. **d**, Timelapse MIPs by cLFM (upper) and csLFM (lower), showing the phenomenon of another neutrophil migrating along vessels and producing multiple retractosomes. **e**, Box plot showing quantified diameter of retractosomes by cLFM and csLFM. The box plot format: center line, median; box limits, lower and upper quartiles; and whiskers, 0th–100th percentiles. *n* = 8 retractosomes were used for statistical analysis. *P* values were calculated by the two-sided paired *t*-test. *P* = 2.25 × 10^−5^. *P* values below 0.05 were considered statistically significant. For quantitative diameter characterization, FWHMs were calculated by measuring the intensity distributions across retractosomes with a Gaussian fit. Scale bars, 10 μm (**a**–**e**,**h**–**k**) and 5 μm (enlarged views in **c**,**d**). MIP, maximum intensity projection.

With the high spatiotemporal resolution, our previously developed sLFM has made great progress in liver imaging, where the formation, division and delivery of migrasomes have been fully studied^31^. Different from migrasomes, retractosomes are newly discovered extracellular vesicles that have smaller sizes, ranging from 50 nm to 250 nm, compared to migrasomes, which is too small for two-photon imaging to resolve clearly, and may play important physiological roles^43^. Although the existence of retractosome structures has been validated in vitro by SDCM with a few images, the function diversity needs to be explored in mammals with timelapse videos. Low phototoxicity, high resolution and high SBR are also required, because retractosomes are small and very susceptible to light. However, in the low-light condition, Poisson-dominated noises originating from the background intensity would overwhelm minute structures, such as retractosomes, and cause artifacts in sLFM (Fig. 4a,b).

To address this problem, we applied csLFM to image the mouse liver with both neutrophil marker (Ly6G) and vessel marker (wheat germ agglutinin (WGA)) under low excitation light for continuous imaging (Supplementary Table 1). By removing the background with better contrast, csLFM enabled a clearer visualization of vascular structures and subcellular dynamics than sLFM and cLFM (Fig. 4a,b and Supplementary Video 4). We then observed that a neutrophil migrated fast along the vessel, and numerous retractosomes were generated from broken-off retraction fibers (Fig. 4c). The intensity profile across a retractosome was plotted to demonstrate the near 300-nm resolution of csLFM in vivo. In another living mouse liver, a similar phenomenon was also observed, in which large quantities of retractosomes were produced and left behind in vessels after the retraction fiber was pulled out at the trailing edge of a neutrophil (Fig. 4d). These retractosomes were distributed as a string of beads, all detected with sub-micron diameters by csLFM, whereas cLFM misidentified them as larger structures spanning several microns (Fig. 4e). It should be noted that the measured diameters by csLFM might be slightly larger than the actual values because the retractosome size is close to the diffraction limit. Nevertheless, csLFM could still accurately preserve the morphology of delicate retractosome structures and distinguish them from the migrasome, which was usually 0.5–2 μm in diameter. Retractosomes were found more in number and smaller in size than migrasomes in mammals. We anticipate that, in the future, csLFM will be further used to investigate how retractosomes or other small vesicles are involved in immune responses.

### High-fidelity high-speed 3D neural recording with reduced crosstalk in vivo

**Fig. 5:**
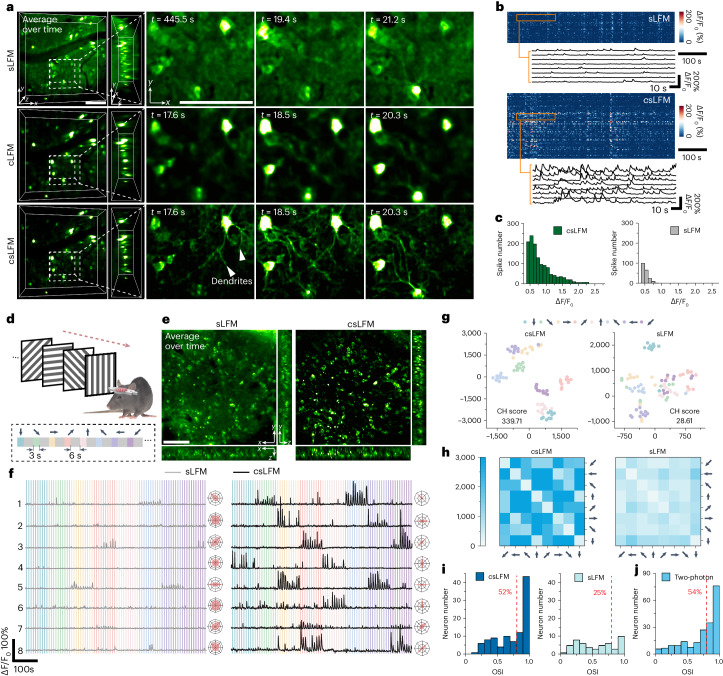
csLFM improves fidelity and neuronal orientation selectivity in high-speed 3D neural imaging in awake mice. **a**, Comparison among sLFM, cLFM and csLFM in a mouse cortex labeled with GCaMP6f at 20 VPS. Displayed volumes are averages over time, and images are enlarged MIPs over the *z* axis. *t* = 0 s in each experiment represents the moment when the corresponding data started to be collected. Frames with similar neural responses are presented for comparison. The neuronal signals were captured at a center imaging depth of approximately 150 μm below the brain surface within an axial coverage of 60 μm. **b**, Temporal traces automatically extracted from **a**. Zoom-in panels show example traces from the same neurons. **c**, Histogram showing comparison of spike amplitudes. *n* represents the number of identified spikes. *n* = 199 for sLFM and *n* = 1,344 for csLFM. **d**, Schematic of visual stimulus with eight-direction moving gratings. **e**, Orthogonal MIPs captured by sLFM and csLFM in the V1 cortex of a mouse expressing GCaMP6f. **f**, Representative temporal traces under the stimuli. Eight identical neurons were analyzed in sLFM and csLFM. The colored blocks indicate the direction of moving grating during the period, as illustrated in **d**. Polar plots for eight directions are also attached to show the tuning curve. **g**–**i**, Comparisons of dimensionality reduction (t-SNE) maps (**g**), distance matrixes (**h**) and OSI distributions (**i**) on the extracted temporal traces. Each point in t-SNE maps corresponds to the neural activity for one direction, and the Calinski–Harabasz (CH) scores are indicated. We set 0.8 as a threshold for high and low OSI, and the proportion of neurons with OSI > 0.8 was used to calculate the percentage. *n* = 51 neurons for sLFM and *n* = 106 for csLFM. The results in **f**–**i** were obtained from four mice in independent experiments. **j**, OSI distributions obtained from a two-photon microscope were used as the reference. Scale bars, 100 μm (**a**,**e**). MIP, maximum intensity projection.

One of the typical applications of LFM is high-speed 3D neural recording. However, tissue scattering and dense fluorescence labeling introduce strong crosstalk contaminations during parallel detection, leading to a fundamental tradeoff between the number of neurons recorded simultaneously and the data fidelity. By increasing the spatial resolution and rejecting the background fluorescence, csLFM reduces the crosstalk while maintaining high 3D imaging speed (Fig. 5a).

To verify the advantages, we constructed an upright csLFM system equipped with a ×25/1.05-NA water immersion objective (Supplementary Fig. 18 and Methods) and observed calcium activities at the cortical layer 2/3 in an awake mouse expressing genetically encoded GCaMP6f (Ai148D). To increase the DOF, the slit size was set as 26 AU for higher angular resolution of 21 × 21 in the upright system^44^. We compared the performance of sLFM and csLFM by imaging the identical region of interest (ROI) at 20 volumes per second (VPS; Supplementary Video 5). The cLFM counterparts were extracted from raw data of csLFM and analyzed. We used the CNMF algorithm^45^ to extract the calcium traces at single-cell level. Given the relatively large size (~10 μm) of neurons and sparse distribution in mice, the functional traces extracted from cLFM results resembled those obtained with csLFM, yet distinguishing subcellular dendritic structures was difficult for cLFM (Supplementary Fig. 19a). Compared to sLFM, though, csLFM exhibited much more identified spike numbers and higher Δ*F*/*F*_*0*_ amplitudes with better contrast (Fig. 5b,c). Applying background subtraction to sLFM data moderately improved the spike amplitude; however, it could not identify the sufficient number of neurons and spikes as in csLFM (Supplementary Fig. 19b,c). Some spikes engulfed by the background are difficult to recover.

To further analyze the data fidelity for neuroscience applications, we recorded the calcium dynamics in primary visual cortex under multiple rounds of visual stimuli with a moving grating (Fig. 5d). It is a classical experiment to measure the tuning properties of neurons responding to visual stimulus, which has been widely verified by electrophysiology and two-photon imaging^46,47^. Except for better contrast in csLFM, we found that the same neurons with a sharp tuning curve in csLFM showed broadened curves in sLFM due to strong background crosstalk (Fig. 5e,f). In addition, many weak responses are flooded in the increased baseline signal in sLFM, which has been well retrieved in csLFM. The inter-class distances of csLFM were larger than those of sLFM during clustering (Fig. 5g,h). We further calculated the orientation selectivity index (OSI) for the neurons (Fig. 5i). Across four mice independently observed by sLFM, only 25% of all visually responsive neurons had orientation tunings larger than 0.8, whereas approximately 52% had OSIs larger than 0.8 in csLFM data on the same mice. The latter OSI distribution was similar to the results obtained by 2D imaging with a two-photon microscope (Fig. 5j and Supplementary Fig. 20), which also accords well with previous studies^48^.

**Fig. 6:**
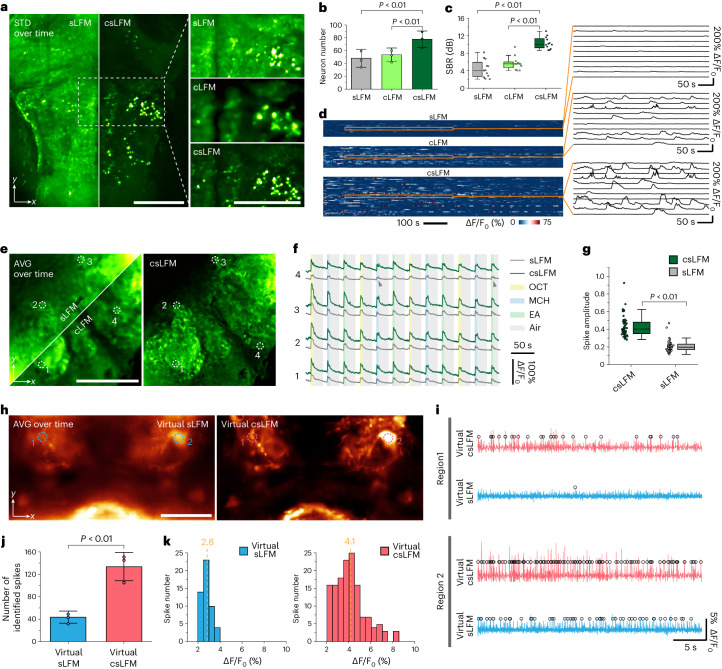
csLFM can be applied in diverse species and indicators with better fidelity. **a**, Comparison among sLFM, cLFM and csLFM in a zebrafish larva labeled with GCaMP6s at 20 VPS. Displayed images are MIPs of STD volumes over time. **b**, Bar chart of detected neuron number. *n* = 3 independent experiments. *P* = 2.39 × 10^−3^ between sLFM and csLFM and *P* = 2.43 × 10^−3^ between cLFM and csLFM. Data are represented as means ± s.d. **c**, Box plot showing SBR comparison. SBR was calculated on the s.d. image where each neuron represents a signal. *n* represents identified neuron number. *n* = 66 for sLFM, *n* = 86 for cLFM and *n* = 158 for csLFM. *P* = 5.36 × 10^−7^ between sLFM and csLFM and *P* = 1.8 9× 10^−8^ between cLFM and csLFM. **d**, Temporal traces extracted from **a** with zoom-in panels. **e**, Comparison among different methods in a *Drosophila* brain labeled with GCaMP7f at 60 VPS. Displayed images are MIPs of the average volumes over time. **f**, Temporal traces extracted from manually selected regions in **g**, with colors implying odor stimuli. Arrows point to spikes almost swamped by sLFM but clearly identified by csLFM. **g**, Box plot showing spike amplitude comparison. *n* = 48 identified spikes and *P* = 4.03 × 10^−22^. **h**, Comparison of dopamine neurons at a depth of approximately 100 μm in *Drosophila* brain (*TH-Gal4×UAS-pAce*) expressing voltage signals, imaged by sLFM and csLFM at 150 VPS. Displayed images are MIPs of the average volumes over time. **i**, Temporal traces extracted from manually selected regions in **h**, with black circles marking the spikes. **j**, Bar chart of detected spike number by different methods. *n* = 3 independent experiments. *P* = 9.98 × 10^−3^. Data are represented as means ± s.d. **k**, Histogram showing spike amplitude. *n* represents identified spike number. *n* = 53 for sLFM and *n* = 143 for csLFM. Format of all box plots: center line, median; box limits, lower and upper quartiles; and whiskers, 0th–100th percentiles excluding outliers. *P* values were all calculated by two-sided paired *t*-test, and *P* < 0.05 was considered statistically significant. Scale bars, 100 μm (**a**,**e**) and 50 μm (**h**). MIP, maximum intensity projection; STD, standard deviation; OCT, 3-octanol; MCH, 4-methylcyclohexanol; EA, ethyl acetate.

Because csLFM does not rely on any sample prior, it can be generally applied to diverse species with different indicators. Next, we recorded timelapse calcium transient of the whole brain in a zebrafish larva expressing genetically encoded GCaMP6s (*huc:GCaMP6s*). The zebrafish brain contains a high density of neuron populations in foreground and background layers with small neuronal size, requiring high resolution to identify single cells clearly. After improving spatial resolution and rejecting out-of-focus light, more neurons were identified with better resolution and higher fidelity in csLFM than in cLFM and sLFM (Fig. 6a–d and Supplementary Video 6). Similarly, we imaged a craniotomized *Drosophila* expressing genetically encoded GCaMP7f (*nsyb-Gal4×UAS-jGCaMP7f*) in vivo at 60 VPS. The *Drosophila* brain is very different from mammalian and zebrafish brains in morphology, and its dense labeling leads to an intense background and low resolution, which is ameliorated by csLFM (Fig. 6e). We gave the *Drosophila* multiple rounds of odor stimuli, to motivate the calcium responses. Approximately two-fold increase in spike amplitude was observed, thereby verifying the effectiveness of csLFM (Fig. 6f,g).

Voltage indicators present more direct measurements of neural activities at higher speed but also pose more challenges for microscopy^49^. Usually, very sparse labeling is used to increase the contrast for the recording of single-trial action potentials in vivo^50^. Here, we performed volumetric voltage imaging in an awake behaving *Drosophila* (*TH-Gal4×UAS-pAce*) at a depth of 100 μm and speed of 150 VPS by labeling almost all dopaminergic neurons densely. Due to the ultrafast voltage transients, the image plane drifting was replaced by the virtual scanning network (Vs-Net) that we proposed previously^50^ to achieve a higher temporal resolution. Compared to sLFM, csLFM reduced the background and improved the image contrast (Fig. 6h and Supplementary Video 7). Notably, some spontaneous voltage potentials drowned by the background in sLFM can be recognized by csLFM with high fidelity (Fig. 6i). csLFM obtained nearly a three-fold increase in identified spike number and 1.5 times enhancement in spike amplitude quantitatively (Fig. 6j,k). With its commendable performance and versatility, csLFM shows tremendous potential as a tool for large-scale neural recording at high fidelity.

## Discussion

In the present study, we combined concepts from line-confocal imaging and sLFM to address the inherent tradeoff between data parallelization and fidelity originating from background fluorescence. Through comprehensive integration spanning wave optics, synchronization of scanning light-sheet illumination and a camera rolling shutter and 3D reconstruction algorithm, csLFM offers a compact solution to retrieve effective signals from severe background and shot noises, and it achieves intravital 3D imaging in mammals with subcellular resolution and low phototoxicity at high fidelity up to camera frame rate.

csLFM stands out as a distinctive technology in comparison to other advanced microscopes. Light-sheet microscopy could effectively eliminate background light with minimal photobleaching by exciting only the in-focus 2D plane, ensuring high-sensitivity recordings during prolonged imaging of transparent samples, such as embryo and cleared tissue^13,51,52^. With very thin illumination sheet such as lattice light-sheet microscopy, light-sheet microscopy can have better axial resolution than csLFM, which is still limited by the missing cone problem. However, light-sheet microscopy faces several constraints in mammalian imaging, such as the tradeoff between sheet thickness and length for wide field of view (FOV) imaging, susceptibility to tissue scattering and more axial scanning required for 3D imaging due to the small DOF as compared to csLFM. Two-photon imaging has an intrinsic advantage in deep tissue observation due to nonlinear excitation with longer wavelength, with broad applications for deep layer neural recording^53^ and innate immune responses^37^. However, the diffraction limit of two-photon microscopy is approximately 500 nm, making it challenging for monitoring behaviors of small organelles, such as the formation processes of retractosomes. Furthermore, similar to confocal microscopy, two-photon microscopy requires sequential scanning of a focal spot, collecting only in-focus photons with unnecessary heat damage to out-of-focus planes. The data throughput of two-photon microscopy is fundamentally limited by the fluorescence decay time and laser repetition rate, which is much smaller than the csLFM with imaging sensors of a large pixel number. In addition, two-photon microscopy usually requires a femtosecond laser, which is more expensive than the compact low-cost system of csLFM. Although csLFM cannot maintain high resolution in deep tissue, such as a depth of approximately 300 μm in mouse cortex, its high-speed, high-resolution, high-fidelity and low-phototoxicity features confer advantages in observing the large-scale behaviors of organelles in mammals and high-throughput recording of functional responses and intercellular interactions.

Several extensions are anticipated to csLFM. First, the snapshot DOF of csLFM can be further enhanced with wavefront coding or multi-focus excitation^54^ up to the working distance of the objective lens. By customizing better objective lenses, the working distance can be further increased—for example, 20 mm for a 0.5-NA objective^55^. Axial scanning^36^ could also be used to increase the axial coverage of current csLFM at the cost of temporal resolution (Supplementary Fig. 21). Second, more accurate modeling of the scattering process in csLFM would be beneficial to increase the penetration depth and enable more quantitative imaging in deep tissue. Longer-wavelength imaging^56^ or multi-photon light-field detection by manipulating the excitation light beam^42^ can also reduce the scattering effect. Third, implanting denoise algorithms^57,58^ into csLFM can further reduce the excitation power, fulfilling the long-term observation of organisms very sensitive to light doses. Fourth, the imaging speed of csLFM is limited mainly by the camera frame rate, which can be increased with smaller ROI or faster cameras. Fifth, as a computational imaging method, csLFM requires more time for iterative reconstruction, which is expected to be further accelerated by deep learning methods^59^.

## Methods

### Experimental setup

#### The inverted system

We made hardware modifications based on the inverted sLFM system reported in our previous work^31^. Our system serves as an add-on to a commercially available inverted microscope (Zeiss, Observer Z1), with the configuration of a ×63/1.4-NA oil immersion objective (Zeiss Plan-Apochromat ×63/1.4 Oil M27). In this way, our csLFM system can be compatible with standard sample holders for wide-field microscopes without any modifications. A scientific complementary metal-oxide-semiconductor (sCMOS) camera (Andor Zyla 4.2 PLUS), which has both global shutter mode and rolling shutter mode, was employed to collect fluorescent signals, with the pixel resolution of 2,048 × 2,048 and the pixel size of 6.5 μm. In global shutter mode, all pixels are simultaneously active during the exposure time. While in rolling shutter mode, a pixel window consisting of only a few adjacent pixel lines is actively exposed at one time and serially moves across the entire image sensor. In the detection path, we inserted a commercial MLA (RPC Photonics MLA-S100-f21) with a pitch size of 100 μm and a focal length of 2,100 μm at the image plane, which were then relayed by a pair of lenses with magnification of 0.845. In this way, each microlens covers exactly 13 × 13 camera pixels, achieving the angular resolution of 13 × 13. A piezoelectric tip and tilt platform (PI S-340 Piezo Tip/Tilt Platform) was used to drift the image plane periodically before the emission light enters the MLA. For cLFM acquisition, the platform was kept unscanned. In the illumination path, the beam output from multi-channel lasers (Coherent, OBIS 405/488/561/640) was first collimated by a convex lens and then modulated by a cylindrical lens (Thorlabs, LJ1363L2-A) into a thin light sheet along the *x–z* plane of the sample. The cylindrical lens played a role in improving laser efficiency. Next, the light-sheet beam passed through an optical slit with the specific width of 325 μm (~11 AU at the conjugate image plane), which sat approximately 100 mm in front of the back focal plane of the cylindrical lens, to generate an axially elongated line-confocal illumination. The offset distance makes the beam almost uniform within a 15-μm axial range in the object space, so the optical slit can act as a beam-shaping device to provide confocality (Supplementary Fig. 2a,b). A pair of relay lens and a *y* axis galvo (Thorlabs, GVS211) were implemented to scan the illumination beam and transmit it from the slit to the conjugated object plane. The multi-color fluorescence imaging was carried out with a multi-channel filter module, including a dichroic mirror (Chroma ZT405/488/561/640rpcv2) and filters (Chroma ZET405/488/561/640xv2 and Chroma ZET405/488/561/640mv2). When acquiring confocal scanning light-field images, the camera, galvanometer, piezoelectric platform and lasers were synchronically triggered using a controlling box (National Instruments, USB-6363) and our customized LabVIEW (2019 version) programs (Supplementary Fig. 1). During the acquisition of one image, a galvo translates the illumination beam, synchronized with the exposure of rolling shutter, beginning at the topmost pixel row and moving across the entire sensor region until the last row. Specifically, we drove the one-dimensional (1D) illumination galvo by a saw-tooth voltage signal and controlled the camera, lasers and piezoelectric platform using step-function voltage signals synchronically. Note that the triggered voltage should be precisely calibrated, so that the center of axially elongated line-confocal illumination is always coincident with the center of rolling window during the acquisition. The maximum beam deflection should exactly correspond to the first and last rows of the camera and the intermediate beam position moved linearly with the angle scanning of galvo. The data shown in Figs. 1–4, Supplementary Figs. 2–8, 10, 12, 13, 15, 16 and 21 and Supplementary Videos 2–4 of this paper were captured by the inverted system.

#### The upright system

We additionally built an upright system for intravital imaging of animals’ brains. We constructed a customized microscope using Thorlabs Cerna series, with a ×25/1.05-NA water immersion objective (Olympus, XLPLN25XWMP2) and an infinity-corrected tube lens (Thorlabs, TTL180-A) to cover a larger field of view. A sCMOS camera (Teledyne Photometrics Kinetix) with larger photosensitive area, faster speed and higher quantum efficiency was used. The camera pixel size is also 6.5 μm. To achieve imaging in deeper tissues, we customized an MLA with the larger pitch size of 136.5 μm and focal length of 2,800 μm, which exactly covers 21 × 21 pixels with the 1:1 relay lens between the MLA and the camera. The more angles we capture, the larger effective DOF can be achieved, and it is more suitable for deep imaging in mammalian brains. Accordingly, we customized another optical slit with larger width of 650 μm (~26 AU at the conjugate image plane) to increase axial coverage. Another relay lens with magnification of 1.64 was attached between the tube lens and the MLA to match NA between the objective and the MLA^44^. The piezoelectric tip and tilt platform in the upright system was replaced with a more affordable one (Core Morrow P33.T2S) that still met precision needs. For cLFM acquisition, the platform was kept unscanned. Other components of the upright system, including lasers, filter modules, cylindrical lens, 1D illumination galvo and the synchronous control procedures, are consistent with the inverted system. The data shown in Figs. 5 and 6, Supplementary Figs. 9, 14, 19 and 20 and Supplementary Videos 5–7 of this paper were captured by the upright system.

Detailed imaging conditions and parameters for all fluorescence experiments, including the fluorescence label, laser, excitation power, line exposure time, total dwell time, imaging speed, system mode, angular resolution, objective and confocality parameters, are illustrated in Supplementary Table 1.

### PSF derivation for csLFM and principle of background suppression

In sLFM^31^, the spatial-angular measurements of an arbitrary 3D point from object space with lateral coordinates of ***p*** = (*p*_*x*_, *p*_*y*_) and axial coordinate of *p*_*z*_ can be represented as

1 $$\begin{array}{l}{W}_{{p}_{z}}({\bf{p}},{{\bf{x}}}_{{\bf{0}}},{\bf{u}})=\displaystyle{\int }_{{\bf{x}}{\prime\prime} }\left\Vert \frac{{e}^{j\frac{2\pi n}{\lambda }{f}_{\mu lens}}}{j\frac{2\pi n}{\lambda }{f}_{\mu lens}}\exp \left(j\frac{\pi n}{\lambda {f}_{\mu lens}}{\Vert {\bf{x}}{\prime\prime} \Vert }_{2}^{2}\right){F}_{\frac{2\pi n}{\lambda {f}_{\mu lens}}{\bf{x}}{\prime\prime} }{\vphantom{\frac{{\bf{x}}}{{d}_{l}}}}\left({U}_{{p}_{3}}({\bf{x}}+{{\bf{x}}}_{{\bf{0}}}-{\bf{p}})\right.\right.\\ \displaystyle \qquad\qquad\qquad\quad\left.\left.\,\cdot\, rect\left(\frac{{\bf{x}}}{{d}_{l}}\right)\right)\cdot rect\left(\frac{{\bf{x}}{\prime\prime} -{\bf{u}}}{{d}_{s}}\right){\vphantom{\frac{{e}^{j\frac{2\pi n}{\lambda }{f}_{\mu lens}}}{j\frac{2\pi n}{\lambda }{f}_{\mu lens}}\exp \left(j\frac{\pi n}{\lambda {f}_{\mu lens}}{\Vert {\bf{x}}{\prime\prime} \Vert }_{2}^{2}\right)}}\right\Vert _{2}^{2}d{\bf{x}}{\prime\prime} ,\end{array}$$

where ***x***_***0***_ = (*x*_0_, *y*_0_) denotes the center position of the microlens and ***x*** = (*x*, *y*) and ***u*** = (*u*, *v*) denote the relative lateral displacement to the center position at the native image plane and the sensor plane, respectively. ${U}_{{p}_{z}}({\bf{x}}-{\bf{p}})$ denotes the complex field at the native image plane derived by the Debye theory; *n* is the refractive index; *λ* is the wavelength of emission light; *d*_*l*_ and *f*_*μlens*_ are the pitch size and focal length of MLA; *d*_*s*_ is sensor pixel size; *rect*(·) is the 2D rectangle function; and *F*(·) is the 2D Fourier transform. Here, we temporarily ignore discrete property of detector pixels and obtain the expression of ${W}_{{p}_{z}}$ in the continuous domain, which can be represented as

2 $$\begin{array}{l}h{s}_{{p}_{z}}({\bf{p}},{{\bf{x}}}_{{\bf{0}}},{\bf{u}})=\displaystyle \Bigg\Vert \frac{{e}^{j\frac{2\pi n}{\lambda }{f}_{\mu lens}}}{j\frac{2\pi n}{\lambda }{f}_{\mu lens}}\exp \left(j\frac{\pi n}{\lambda {f}_{\mu lens}}{\Vert {\bf{u}}\Vert }_{2}^{2}\right){F}_{\frac{2\pi n}{\lambda {f}_{\mu lens}}{\bf{u}}}{\vphantom{\frac{{\bf{x}}}{{d}_{l}}}}\left({U}_{{p}_{3}}({\bf{x}}+{{\bf{x}}}_{{\bf{0}}}-{\bf{p}})\right.\\ \displaystyle \qquad\qquad\qquad\,\cdot \, rect\left.\left(\frac{{\bf{x}}}{{d}_{l}}\right)\right)\Bigg\Vert _{2}^{2}.\end{array}$$

Note that ***u*** is limited by the microlens pitch, and ***x***_***0***_ is a discrete variable with a period of microlens diameter. So, ***x***_***0***_ + **u** can represent every point at the sensor plane, which is named sensor coordinates—that is, $\tilde{{\bf{x}}}=(\tilde{x},\tilde{y})={{\bf{x}}}_{{\bf{0}}}+{\bf{u}}$. $h{s}_{{p}_{z}}({\bf{p}},{{\bf{x}}}_{{\bf{0}}},{\bf{u}})$ can be represented as $h{s}_{{p}_{z}}({\bf{p}},{{\bf{x}}}_{{\bf{0}}}+{\bf{u}})$ $=h{s}_{{p}_{z}}({\bf{p}},\tilde{{\bf{x}}})$.

The optical slit is not exactly at the back focal plane of the cylindrical lens in csLFM setup. We thereby assume that the beam passing through the slit has a uniform pattern with a finite size. As shown in Supplementary Fig. 2b, the axially elongated line-confocal illumination can be approximated as a collimated sheet and represented by a rectangle function at each *p*_*z*_ plane. The illumination is scanned along the *p*_*y*_ axis while rolling shutter is sliding on the $\tilde{y}$ axis (Supplementary Fig. 2c). When the width of illumination slit *w*_*s*_ and the row height of rolling shutter *h*_*r*_ are known, the imaging process can be modeled as an integration over the illumination scanning (Fig. 1b). Therefore, the continuous form of csLFM PSF can be represented as

3 $$\begin{array}{c}h{s{\prime} }_{{p}_{z}}({\bf{p}},{\tilde{\bf{x}}}{\boldsymbol{,}}{w}_{s},{h}_{r})=\mathop{\displaystyle\int }\limits_{-\infty }^{+\infty }\left[h{s}_{{p}_{z}}({\bf{p}},{\tilde{\bf{x}}})\cdot rect\left(\frac{{p{\prime} }_{y}-{p}_{y}}{{w}_{s}/M}\right)\cdot rect\left(\frac{\tilde{y}-{p{\prime} }_{y}\cdot M}{{h}_{r}}\right)\right]d{p{\prime} }_{y}\\ =h{s}_{{p}_{z}}({\bf{p}},{\tilde{\bf{x}}})\mathop{\displaystyle\int }\limits_{-\infty }^{+\infty }rect\left(\frac{{p{\prime} }_{y}-{p}_{y}}{{w}_{s}/M}\right)\cdot rect\left(\frac{\tilde{y}-{p{\prime} }_{y}\cdot M}{{h}_{r}}\right)d{p{\prime} }_{y},\end{array}$$

where *M* is magnification of the objective lens. Normally, in line confocal microscopy, the illumination slit and detector could maintain a conjugate relationship to enhance the confocal effect^60^. Thus, we assume that *w*_*s*_ = *h*_*r*_, and equation (3) could be represented as

4 $$\begin{array}{l}h{s{\prime} }_{{p}_{z}}({\bf{p}},{\tilde{\bf{x}}}{\boldsymbol{,}}{w}_{s})\,^{\displaystyle\underline{\underline{{p{\prime\prime} }_{y}={p{\prime} }_{y}-{p}_{y}}}}\,h{s}_{{p}_{z}}({\bf{p}},{\tilde{\bf{x}}})\mathop{\displaystyle\int }\limits_{-\infty }^{+\infty }rect\left(\frac{{p{\prime\prime} }_{y}\cdot M}{{w}_{s}}\right)\\\qquad\qquad\qquad\cdot rect\left(\frac{(\tilde{y}-{p}_{y}\cdot M)-{p{\prime\prime} }_{y}\,\cdot \, M}{{w}_{s}}\right)d{p{\prime\prime} }_{y}\\\qquad\qquad\qquad =h{s}_{{p}_{z}}({\bf{p}},{\tilde{\bf{x}}})\cdot tri\left(\frac{\tilde{y}-{p}_{y}\cdot M}{{w}_{s}}\right)\\\qquad\qquad\qquad =h{s}_{{p}_{z}}({\bf{p}},{\tilde{\bf{x}}})\cdot {g}_{s}({\bf{p}},{\tilde{\bf{x}}}),\end{array}$$

where *tri*(*x*) is a 1D triangle function, $$tri(x)=\left\{\begin{array}{lc}1-|x|, & |x| < 1\\ 0, & |x|\ge 1\end{array}\right.$$, and ${g}_{s}({\bf{p}},\tilde{{\bf{x}}})=tri\left(\frac{\tilde{y}-{p}_{y}\cdot M}{{w}_{s}}\right)$ is called as the confocal modulation function at the sensor plane.

Then, we go into how the confocal modulation function affects the PSFs in spatial-angular domain. As mentioned in our previous work^31^, transforming the original PSFs into spatial-angular PSFs is carried out by pixel realignment operator in the discrete domain. While in the continuous domain, the realignment process can be considered as a dimension exchange between ***p*** and ***u***, which is derived as

5 $$h{s}_{{p}_{z}}({\bf{p}},{\tilde{\bf{x}}})=h{s}_{{p}_{z}}({\bf{p}},{{\bf{x}}}_{{\bf{0}}}+{\bf{u}})=h{p}_{{p}_{z}}({{\bf{x}}}_{{\bf{0}}}-{\bf{p}}\cdot M,{\bf{u}})=h{p}_{{p}_{z}}(\bar{{\bf{x}}},{\bf{u}}),$$

where $h{p}_{{p}_{z}}$ denotes the spatial-angular PSFs, and $\bar{{\bf{x}}}=(\bar{x},\bar{y})$ $=({x}_{0}-{p}_{x}\cdot M,{y}_{0}-{p}_{y}\cdot M)$ is spatial coordinates in spatial-angular domain. The modulation function is also rewritten as

6 $$\begin{array}{c}{g}_{s}({\bf{p}},{\tilde{\bf{x}}})=tri\left(\frac{\tilde{y}-{p}_{y}\cdot M}{{w}_{s}}\right)\\ =tri\left(\frac{{y}_{0}+v-{p}_{y}\cdot M}{{w}_{s}}\right)\\ =tri\left(\frac{\bar{y}+v}{{w}_{s}}\right)\\ ={g}_{p}(\bar{{\bf{x}}},{\bf{u}}),\end{array}$$

where ${g}_{p}(\bar{{\bf{x}}},{\bf{u}})$ is the modulation function in spatial-angular domain. Additionally, because the slit size *w*_*s*_ is much larger than the absolute value of offset |*v*|, the effect of the offset can be neglected, and the modulation function can be simplified as ${g}_{p}(\bar{{\bf{x}}},{\bf{u}})=tri\left(\frac{\bar{y}}{{w}_{s}}\right)$. Hence, the spatial-angular PSF of csLFM can be represented as

7 $$h{p{\prime} }_{{p}_{z}}(\bar{{\bf{x}}},{\bf{u}},{w}_{s})=h{p}_{{p}_{z}}(\bar{{\bf{x}}},{\bf{u}})\cdot tri\left(\frac{\bar{y}}{{w}_{s}}\right).$$

After the confocal modulation, the background in angular views that deviate from origin of $\bar{y}$ axis is attenuated, whereas the paraxial views remain unchanged (Supplementary Fig. 2d). As the distance from the focal plane increases, the PSF pattern of sLFM becomes larger and contains more background. When confocal module is imposed, csLFM prominently suppresses the energy from the out-of-focus light while maintaining the energy near the focal plane (Fig. 1c).

It should be explained that the capability of csLFM on background suppression varies in different spatial-angular components. On account of the modulation function being uniform along the $\bar{x}$ axis, we only consider the *p*_*z*_ and $\bar{y}$ dimensions here. The dip angle of the PSF increases obviously as |*v*| increases, thus causing the pattern to deviate more from the origin of $\bar{y}$ axis and the background suppression to become more substantial. For quantitative analysis, we define the energy of sLFM PSF ${E}_{{p}_{z}}({\bf{u}})$ and the energy of csLFM PSF ${E{\prime} }_{{p}_{z}}({\bf{u}},{w}_{s})$:

8 $${E}_{{p}_{z}}({\bf{u}})=\mathop{\iint }\limits_{\varPhi }h{p}_{{p}_{z}}({\bf{u}},\bar{{\bf{x}}})d\bar{x}d\bar{y},$$

9 $${E{\prime} }_{{p}_{z}}({\bf{u}},{w}_{s})=\mathop{\iint }\limits_{\varPhi }h{p{\prime} }_{{p}_{z}}({\bf{u}},\bar{{\bf{x}}},{w}_{s})d\bar{x}d\bar{y},$$

where Φ denotes the whole $\bar{{\bf{x}}}$ plane. The normalized energy of csLFM PSF ${\bar{E{\prime} }}_{{p}_{z}}({\bf{u}},{w}_{s})$ can be defined as

10 $${\bar{E{\prime} }}_{{p}_{z}}({\bf{u}},{w}_{s})=\frac{1}{{E}_{0}({\bf{u}})}{E{\prime} }_{{p}_{z}}({\bf{u}},{w}_{s})=\frac{1}{{E}_{0}({\bf{u}})}\mathop{\iint }\limits_{\varPhi }h{p{\prime} }_{{p}_{z}}({\bf{u}},\bar{{\bf{x}}},{w}_{s})d\bar{x}d\bar{y},$$

which embodies the background suppression ability of csLFM. With the increase of the |*p*_*z*_|, ${\bar{E}{\prime} }_{{p}_{z}}$ decays rapidly, and the decay speed is positively correlated with the |*v*| (Supplementary Fig. 2e). The effect of *u* to the background rejection is much less prominent compared to *v*, forming an ellipse-like pattern (Supplementary Fig. 2f,g).

### Design of confocality parameters

In our csLFM implementation, confocality parameters consist mainly of the illumination slit size and the rolling row height. The former can be adjusted by the optical element, and the latter can be set digitally using camera software development kits (SDKs). The confocal performance is determined by the minimum value of the two parameters. For simplicity, we set the slit size the same as the height of rolling shutter. The sCMOS we used, both the Andor Zyla 4.2 PLUS camera (implemented in the inverted system) and the Teledyne Photometrics Kinetix camera (implemented in the upright system), can achieve the desired value of rolling row height by adjusting the exposure time of each pixel row and the exposure starting interval between two adjacent pixel rows (Supplementary Fig. 1a). Of course, these values cannot be adjusted arbitrarily, because the scanning speed of the rolling shutter is limited. When the rolling row height is given, the camera SDKs can automatically feed back the corresponding value range of exposure time and imaging frame rate, based on the scanning speed that can be achieved. In practice, we set the exposure time and frame rate flexibly according to the different imaging requirements.

In parameter selection, the ability to reduce background, photon efficiency and the achievable axial coverage are the major considerations. On the one hand, as the slit size increases, the ability to remove background becomes weaker, and the axial coverage and photon efficiency become larger. On the other hand, as the slit size decreases, the background removal ability becomes stronger, but the axial coverage and photon efficiency become smaller. In the inverted system with angular resolution of 13 × 13, we set the slit size as 11 AU, achieving the axial coverage of over 15 µm, 80% photon efficiency and decent background-rejected capability simultaneously, as analyzed in Supplementary Figs. 3 and 4. While in the upright system, because the angular resolution is increased to 21 × 21, the NA of each sub-aperture becomes smaller, so that the system fundamentally has a larger DOF range, as analyzed in Supplementary Fig. 18. We choose the illumination slit with width of approximately 26 AU for the upright system, to meet the requirements of increased axial coverage, 80% photon efficiency and background-rejected capability in brains. The selected width of rolling shutter also approximates the sub-aperture AU (defined by $\frac{1.22\cdot \lambda \cdot M}{N{A}_{sub-aperture}}$, where *NA*_*sub-aperture*_ is the sub-aperture NA after pupil segmentation by MLA, and *M* is the magnification of the whole optical system), which is also in agreement with the published paper^60^.

### Comparison of csLFM with sLFM and cLFM

To compare the performance of csLFM and sLFM, we need to toggle the two imaging configurations in one system. As described in PSF derivation, the narrow window height and the thin axially elongated line-confocal illumination work together to gain optical confocality. sLFM imaging can be realized by setting the global shutter mode, increasing window height and removing optical slit or all of them. In practice, the switching between csLFM and sLFM was achieved by alternately setting rolling shutter mode to achieve a narrower and a broader exposure window, respectively, for the convenience without any modifications in hardware system. When the camera window height is set to 2,048 rows (~500 AU) that cannot effectively function as a confocal slit, the rolling shutter approximates the global shutter. In this way, at least half of all pixel rows are kept exposed at any given moment, allowing almost all emitted fluorescence to be collected without blocking, which is equivalent to traditional sLFM.

For static specimens, we captured single-frame data using sLFM and csLFM, respectively. For most biological experiments in living animals, we first collected sLFM and csLFM data at *t* = 0 min for comparison and then captured timelapse data to record biological phenomena using csLFM only. For neural imaging in mice, *Drosophila* and zebrafish, we captured two timelapse data segments using sLFM and csLFM, respectively. For comparison between sLFM and csLFM, the total dwell time of two modes, measured from the central moment of exposure in the first row to the central moment of exposure in the last row, was designed to be similar (Supplementary Table 1).

The raw light-field data of cLFM were extracted from the csLFM data at one specific scanning position, whereby the pairs of csLFM and cLFM data were simultaneously acquired, which was intrinsically well matched and could be directly compared over time. The experimental parameters of cLFM were consistent with those of csLFM except for scanning number (Supplementary Table 1). For 3D reconstruction of cLFM data, a cubic interpolation was firstly used to increase the spatial sampling rate to the same as that of csLFM, followed by a deconvolution operator using iterative tomography^31^.

### Bead phantom preparation for SBR characterization

We made two tissue-mimicking phantoms. One was with 0.0005% 0.5-μm fluorescence beads (Thermo Fisher Scientific, FluoSpheres, carboxylate-modified microspheres, F8813), 1% intralipid (Absin 68890-65-3) and 1% agarose. Another one was with 0.0005% 0.5-μm fluorescence beads, 10% intralipid and 1% agarose. The mixtures were placed in a glass-bottom dish (NEST Scientific Glass Bottom Cell Culture Dish, 801001). The 0.5-μm fluorescence beads were randomly distributed in the mixture of agarose and intralipid and experimentally imaged by sLFM and csLFM with a ×63/1.4-NA oil immersion objective. During imaging, the objective lens was electronically controlled to move up and down with an axial step of 20 μm using MicroManager software (version 1.4.21). For SBR calculation, the maximum intensity of the bead was viewed as the signal, and the mean intensity of regions without beads was viewed as the background, as illustrated in Supplementary Fig. 4b.

### Bead preparation for resolution characterization

We made a mixture of 1 μl of 1% 0.1-μm fluorescence beads (Thermo Fisher Scientific, TetraSpeck Microspheres, T7279) and 1 ml of 1% agarose and placed it in a glass-bottom dish (NEST Scientific Glass Bottom Cell Culture Dish, 801001). The 0.1-μm fluorescence beads were randomly distributed in the agarose and experimentally imaged by cLFM and csLFM with a ×63/1.4-NA oil immersion objective. For resolution evaluation, we wrote a customized MATLAB program to measure FWHM of the cross-section profile for each bead laterally and axially.

### Mouse brain slice preparation

Thy1-YFP-H transgenic mice (The Jackson Laboratory, 003782, male) were used for the preparation of brain slices. A male mouse was first perfused transcardially with 50 ml of 0.01 M PBS and then perfused transcardially with 25 ml of 4% paraformaldehyde (PFA) that was dissolved in 0.01 M PBS. Next, we harvested the brain and placed it in 4% PFA overnight at the constant temperature of 4 °C. A vibratome (VT1200 S, Leica) was used to cut the brain into 300-μm-thick slices. Finally, the slices were sealed in antifade solution (Applygen Technologies, C1210) for imaging. The brain slices were only used as a dense sample for verifying the imaging performance of csLFM.

### Zebrafish experiments

For whole-brain calcium imaging, *Tg(huc:GCaMP6s)* transgenic zebrafish embryos were collected and kept at 28.5 °C in Holtfreter’s solution. At 4 d after fertilization, the zebrafish larvae were mounted in 1% of low-melting-point agarose for calcium imaging at 26–27 °C.

### Mouse experiments

#### Immune imaging

The mice were male and wild-type (C57BL6/J, 7–8 weeks). Mice were housed with water and food available ad libitum under a 12-h light/dark cycle. For labeling of the immune cells, 10 μl of Ly6G (BioLegend, 127607, 10% dilution), CD11c (BioLegend, 117307, 10% dilution), F4/80 (BioLegend, 157313, 10% dilution), CD3 (BioLegend, 100235, 10% dilution), NK1.1 (BioLegend, 108707, 10% dilution) and WGA (Thermo Fisher Scientific, W32466, 10% dilution) were injected into mice by intravenous injection. After 20 min, anesthesia was induced in mice by intraperitoneal injection of Avertin (375 mg kg^−1^). Then, the spleens and livers were dissected to expose for imaging. A syringe was attached to the head of the mice for the subsequent injection of anesthetic. During imaging, a 37 °C body temperature maintenance instrument (RWD Life Science, ThermoStar Homeothermic Monitoring System) maintained the anaesthetized mice in their normal physiological state.

#### Brain imaging

For vascular imaging, the wild-type mouse (C57BL6/J, male, 7–8 weeks) was housed with water and food available ad libitum under a 12-h light/dark cycle. A 7-mm craniotomy was made, and a cranial window was implanted in the mouse. After 2 weeks of recovery, the mouse was intravenously injected with 10 μl of AF647 dye (Thermo Fisher Scientific, A32733, 10% dilution) to label the vascular structures. Then, the mouse was head fixed and imaged by sLFM and csLFM, respectively, at different axial positions. For neural imaging, the transgenic mice (Ai148D, male, 7–8 weeks) were housed with water and food available ad libitum under a 12-h light/dark cycle. The ambient temperature was set to 22 °C, and the relative humidity was kept at 50%. A 7-mm craniotomy was made, and a cranial window was implanted in the mice. After 2 weeks of recovery, the mice were head fixed and imaged by sLFM, cLFM and csLFM, respectively. To observe the visual response, we generated moving grating stimuli along eight directions at 45° apart using the Psychophysics Toolbox^61^ on MATLAB R2021a. The gratings were presented with a commercial LCD monitor and a blue-colored glass filter, placed approximately 100 mm in front of the right eye of the mouse. Then, we used sLFM, csLFM and two-photon microscopy to record the neural activities in the primary visual area (V1) of the left cerebral cortex. During the imaging, a 3-s moving sinusoidal grating (0.02 cycles per degree) followed by a 6-s blank period (uniform gray at mean luminance) were applied as a stimuli trial. For each direction, we conducted 10 trials continuously for one direction and then quickly switched to the next direction. For preparation of traumatic brain injury model, CX3CR1-GFP transgenic mice (The Jackson Laboratory, 008451, male, 8–12 weeks) were used. Then, 10 μl of Ly6G (BioLegend, 164503) was injected into the mice by intravenous injection to label the neutrophils. The closed-head injury was performed by weight drop^62^. Skull thinning was completed by scraping the cranial surface with a micro-surgical blade. Skull thickness was reduced to an approximately 30-μm thin layer for imaging.

### *Drosophila* experiments

The *Drosophila* strain (*TH-Gal4* > *UAS-pAce*) labeled with voltage indicators in all dopaminergic neurons and (*nsyb-gal4×UAS-jGCaMP7f*) labeled with calcium indicators in all neurons were provided by the Schnitzer laboratory at Stanford University and the Zhong laboratory at Tsinghua University. Flies were raised on standard cornmeal medium with a 12-h light/dark cycle at 23 °C and 60% humidity and housed in mixed male/female vials. Three- to eight-day-old female flies were selected for brain imaging. To prepare for imaging, flies were anesthetized on ice and mounted in a 3D-printed plastic disk with free movement of the legs. Then, the posterior head capsules were opened using sharp forceps (Dumont, 5SF) at room temperature in fresh saline (103 mM NaCl, 3 mM KCl, 5 mM TES, 1.5 mM CaCl_2_, 4 mM MgCl_2_, 26 mM NaHCO_3_, 1 mM NaH_2_PO_4_, 8 mM trehalose and 10 mM glucose (pH 7.2), bubbled with 95% O_2_ and 50% CO_2_). The fat body and air sac were also removed carefully. Next, to minimize brain movement^63^, UV glue was also added around the proboscis, and the M16 muscle was removed. After the surgery, the *Drosophila* were placed under the objective lens for imaging.

### Neural activity extraction

For calcium analysis in Figs. 5 and 6, we used the CNMF algorithm^45^ to obtain neuron segmentations and temporal traces for the mouse and zebrafish data, and we manually selected several ROIs as labeled to obtain temporal traces for the *Drosophila* data. The temporal traces were calculated by Δ*F*/*F*_*0*_ = (*F* *−* *F*_*0*_*)* / *F*_*0*_, where *F*_*0*_ is the mean fluorescence in the ROI averaged over the entire time series, and *F* is the averaged intensity of the ROI. The neural spikes were identified as the local peaks that surpassed a threshold value (40%). The amplitude of each spike was calculated as the absolute value of its peak value. The orientation-selective response (polar plots) of each trace was calculated by averaging Δ*F*/*F*_*0*_ during the stimuli for different orientations. For neuronal orientation selectivity analysis, neurons with high visual response (*R* > 3) were considered, where *R* was defined as the ratio of the average Δ*F*/*F*_*0*_ with stimuli to the average Δ*F*/*F*_*0*_ without stimuli. The OSI was calculated as (*R*_*pref*_ − *R*_*orth*_) / (*R*_*pref*_ + *R*_*orth*_)^64^, where *R*_*pref*_ is the maximum orientation-selective response, and *R*_*orth*_ is the mean response with the orientations orthogonal to the *R*_*pref*_. For the t-distributed stochastic neighbor embedding (t-SNE) analysis, we used the MATLAB built-in ‘tsne.m’ function.

For voltage analysis in Fig. 6h–k, we manually selected several ROIs as labeled and obtained Δ*F*/*F*_*0*_ temporal traces followed by a wavelet-based denoising. The neural spikes were identified as the local peaks that surpassed a threshold value (2%) after the Δ*F*/*F*_*0*_ curve subtracts its median-filtered (130-ms window) version and were visualized by the shining color in Supplementary Video 7. The amplitude of each spike was calculated as the absolute value of its peak value. The video of subframe voltage propagation was made with quadratic spline interpolation^65^.

### Ethics statement

This work was carried out with all relevant ethical regulations for animal research. All biological experiments were conducted with ethical approval from the Animal Care and Use Committee of Tsinghua University.

### Performance metrics

To demonstrate the background reduction by csLFM, we used SBR for metric evaluation. SBR was defined as the 10-fold logarithmic ratio between signal (maximum intensity of selected ROIs) and background (average intensity of regions without signals), as described in the following formula:

$$SBR=10{\log }_{10}\left(\frac{\max (are{a}_{S})}{{\rm{mean}}(are{a}_{B})}\right),$$

where *area*_*S*_ represented the signal region, and *area*_*B*_ represented the background region. When the signal is equal to the background, the SBR is 0 dB. The SNR and structural similarity index measure (SSIM) were used to evaluate the reconstruction performance of csLFM. SNR was calculated as

$$SNR=10{\log }_{10}\frac{{\Vert X\Vert }_{2}^{2}}{{\Vert X-Y\Vert }_{2}^{2}},$$

where *X* is the ground truth, and *Y* is the reconstruction result by csLFM. The SSIM was calculated by the following formula:

$$SSIM=\frac{\left(2{\mu }_{X}{\mu }_{Y}+{(0.01\cdot L)}^{2}\right)\left(2{\sigma }_{XY}+{(0.03\cdot L)}^{2}\right)}{\left({\mu }_{X}^{2}+{\mu }_{Y}^{2}+{(0.01\cdot L)}^{2}\right)\left({\sigma }_{X}^{2}+{\sigma }_{Y}^{2}+{(0.03\cdot L)}^{2}\right)},$$

where *μ*_*X*_ and *μ*_*Y*_ are average values of ground truth and reconstruction, and *σ*_*X*_, *σ*_*Y*_ and *σ*_*XY*_ are the corresponding standard deviations and covariance. The data were normalized by their maximum value, and *L* is the maximum value of *X*. The SSIM indices were calculated on 3D images with 3D local Gaussian kernels.

To evaluate the dispersion between data clusters, we used Calinski–Harabasz score, intra-class distance and inter-class distance for metric evaluation. The Calinski–Harabasz score was calculated by the following formula:

$$s=\frac{S{S}_{B}}{S{S}_{W}}\cdot \frac{N-k}{k-1}=\frac{\mathop{\sum }\nolimits_{q=1}^{k}{N}_{q}d\left({{\bf{c}}}_{q}-{{\bf{c}}}_{E},{{\bf{c}}}_{q}-{{\bf{c}}}_{E}\right)}{\mathop{\sum }\nolimits_{q=1}^{k}\mathop{\sum }\nolimits_{n=1}^{{N}_{q}}d\left({{\bf{X}}}_{n}^{(q)}-{{\bf{c}}}_{q},{{\bf{X}}}_{n}^{(q)}-{{\bf{c}}}_{q}\right)}\cdot \frac{N-k}{k-1},$$

where Ω*q* is the *q-*th class; *SS*_*B*_ is the inter-class variance; *SS*_*W*_ is the intra-class variance; *N* is the total sample size; *N*_*q*_ is the sample size of Ω_*q*_; *k* is the number of classes; ${{\bf{X}}}_{n}^{(q)}$ is a feature vector of the *n-*th sample in Ω_*q*_; **c**_*E*_ is the mean of all samples; **c**_*q*_ is the mean of all samples in Ω_*q*_; and *d*(·) is the Euclidean distance between two vectors. The intra-class distance and inter-class distance was calculated, respectively, as

$$d\left({\varOmega }_{q},{\varOmega }_{q}\right)=\frac{1}{{N}_{q}^{2}}\mathop{\sum }\limits_{k=1}^{{N}_{q}}\mathop{\sum }\limits_{l=1}^{{N}_{q}}d\left({{\bf{X}}}_{k}^{(q)},{{\bf{X}}}_{l}^{(q)}\right),$$

$$d\left({\varOmega }_{q},{\varOmega }_{p}\right)=\frac{1}{{N}_{q}{N}_{p}}\mathop{\sum }\limits_{k=1}^{{N}_{q}}\mathop{\sum }\limits_{l=1}^{{N}_{p}}d\left({{\bf{X}}}_{k}^{(q)},{{\bf{X}}}_{l}^{(p)}\right),$$

The intra-class distances together with inter-class distances formed a distance matrix between classes, as shown in Fig. 5h.

### Data analysis

All data processing and analysis were accomplished with customized MATLAB (MathWorks, MATLAB 2019a) scripts. The hardware synchronization was controlled with an NI-USB-6363 box and our customized LabVIEW program. The 3D rendering of the volumes was performed by Imaris (Imaris 9.0.1 software) or Voltex modules in Amira (Thermo Fisher Scientific, Amira 2019). The 3D tracking of immune cells in the spleen was carried out automatically using Imaris. For the analysis of interaction times between NK cells and macrophages, we first conducted tracking and numbering separately for channels of NK cells and macrophages. The NK cells and macrophages were then segmented in each channel. Next, the count of overlapping structures between the two channels was identified as the number of contacting cell pairs. The interaction times at *t* = *t*_0_ were considered as the cumulative values from 0 to *t*_0_. If the *i-*th NK cell and the *j-*th macrophage both interacted in consecutive two frames, the interaction times would not be cumulated. For the analysis of interaction frequency between NK cells and macrophages, NK cells and macrophages were first segmented in each channel. Then, the overlap between the two channels was calculated as a binary mask at each frame. This binary mask was next applied separately to the segmentations of the two channels, resulting in the respective counts of interacting cells in each channel. The sum of these counts provided the interacting cell number. The total cell number was determined on the segmentations in two channels. The frequency was finally returned as the ratio of the number of cells that made interactions to the total cell number. For the analysis of the number of cell pairs between dendritic cells and T cells, the cells were first segmented in each channel, and then the count of overlapping structures between the two channels was returned as the number of contacting cell pairs. For the analysis of retraction fibers, the detection of fibers generated from immune cells was carried out using the ridge detection plug-in of ImageJ software (version 1.51) automatically. Fiber structures with the width of 0.2–1 μm and the length of more than 3 μm were identified, and invalid results were also sifted out manually. For the analysis of the average fiber number produced by each cell pair, fibers that are not generated in cell contact were eliminated manually.

### Reporting summary

Further information on research design is available in the Nature Portfolio Reporting Summary linked to this article.

## Online content

Any methods, additional references, Nature Portfolio reporting summaries, source data, extended data, supplementary information, acknowledgements, peer review information; details of author contributions and competing interests; and statements of data and code availability are available at 10.1038/s41587-024-02249-5.

## Supplementary information

Supplementary Information Supplementary Figs. 1–21, Supplementary Table 1 and titles of Supplementary Videos 1–7.

Reporting Summary

Supplementary Video 1 Illustrations of csLFM compared to traditional sLFM.

Supplementary Video 2 csLFM reveals the delivery of migrasome between NK cells and macrophages in mammals.

Supplementary Video 3 csLFM reveals the interaction of dendritic cells and T cells through generating and elongating retraction fibers in vivo.

Supplementary Video 4 csLFM reveals retractosome formation in mammals with low excitation light.

Supplementary Video 5 csLFM achieves improved resolution and contrast in recording of neural activity in mammals at 20 VPS.

Supplementary Video 6 csLFM obtains better performance in the whole brain of zebrafish larvae at 20 VPS.

Supplementary Video 7 csLFM realizes high-speed volumetric voltage imaging at 150 VPS with reduced background and enhanced spike resolvability in a *Drosophila* brain.

## Data Availability

Data used for comparisons between sLFM and csLFM are publicly available on Zenodo (10.5281/zenodo.8198063)^66^ and GitHub (https://github.com/THU-IBCS/csLFM-master)^67^.

## Code availability

All relevant codes for csLFM are available on Zenodo (10.5281/zenodo.8198063)^66^ and GitHub (https://github.com/THU-IBCS/csLFM-master)^67^.
