# Supplementary material for: Long-term intravital subcellular imaging with confocal scanning light-field microscopy
Source: Nat Biotechnol. 2024 May 27;43(4):569–80. doi: 10.1038/s41587-024-02249-5 (PMC11994454; doi:10.1038/s41587-024-02249-5)
Supplement: Supplementary file 1 — Supplementary Figs. 1–21, Supplementary Table 1 and titles of Supplementary Videos 1–7. [file 41587_2024_2249_MOESM1_ESM.pdf]

nature biotechnology

Supplementary information https://doi.org/10.1038/s41587-024-02249-5

Long-term intravital subcellular imaging with confocal scanning light-field microscopy

In the format provided by the authors and unedited

Supplementary Information

Long-term intravital subcellular imaging with confocal scanning light-field microscopy

Zhi Lu, Siqing Zuo, Minghui Shi, Jiaqi Fan, Jingyu Xie, Guihua Xiao, Li Yu, Jiamin Wu, Qionghai Dai

I. Supplementary Figures

**Supplementary Figure 1**	Illustration of rolling shutter mechanism and csLFM synchronization
**Supplementary Figure 2**	Illustrations of axially elongated line-confocal illumination and spatial-angular PSF modulation in csLFM
**Supplementary Figure 3**	Numerical analysis of the influence of different slit sizes on axial coverage and reconstruction performance
**Supplementary Figure 4**	Numerical analysis of the influence of different slit sizes on SBR and photon efficiency
**Supplementary Figure 5**	Numerical analysis of the imaging performance of sLFM and csLFM with increasing background levels
**Supplementary Figure 6**	Numerical analysis of optical sectioning capability and axial performance of sLFM and csLFM with increasing background levels
**Supplementary Figure 7**	Numerical analysis of the influence of shot noise in csLFM with increasing background levels
**Supplementary Figure 8**	Numerical analysis of digital adaptive optics (DAO) accuracy in csLFM with increasing background levels
**Supplementary Figure 9**	Motion artefacts correction for csLFM imaging
**Supplementary Figure 10**	Experimental SBR characterization in different angular measurements of intralipid-based mixture
**Supplementary Figure 11**	Experimental evaluation of SBR and resolution with different penetration depths in a mouse brain *in vivo*

**Supplementary Figure 12**	Experimental comparisons of a 300-μm-thick Thy1-YFP mouse brain slice imaged by sLFM, sLFM with multiscale model and csLFM
**Supplementary Figure 13**	Experimental analysis of the background-suppressed performance of csLFM and sLFM with direct background subtraction
**Supplementary Figure 14**	Experimental comparisons of a 300-μm-thick Thy1-YFP mouse brain slice imaged by csLFM, SDCM and two-photon microscopy
**Supplementary Figure 15**	Resolution characterization of cLFM and csLFM in 3D fluorescence imaging
**Supplementary Figure 16**	Photon efficiency analysis of csLFM in imaging thin samples
**Supplementary Figure 17**	Formation of retraction fibers and migrasomes in mammals validated by two-photon synthetic aperture microscopy (2pSAM)
**Supplementary Figure 18**	Numerical analysis of the influence of different slit sizes in the upright csLFM system
**Supplementary Figure 19**	Detailed comparison of neural imaging in mouse cortex between cLFM, sLFM and csLFM
**Supplementary Figure 20**	Comparison of neural recordings under visual stimuli between csLFM and two-photon microscopy
**Supplementary Figure 21**	Extended depth of field of csLFM with axial scanning

II. Supplementary Tables

**Supplementary Table 1**	Imaging parameters for all fluorescence experiments

III. Supplementary Videos

**Supplementary Video 1**	Illustrations of csLFM compared to traditional sLFM
**Supplementary Video 2**	csLFM reveals the delivery of migrasome between natural killer (NK) cells and macrophages in mammals

**Supplementary Video 3**	csLFM reveals the interaction of dendritic cells and T cell through generating and elongating retraction fibers *in vivo*
**Supplementary Video 4**	csLFM reveals retractosome formation in mammals with low excitation light
**Supplementary Video 5**	csLFM achieves improved resolution and contrast in recording of neural activity in mammals at 20 VPS
**Supplementary Video 6**	csLFM obtains better performance in the whole brain of zebrafish larvae at 20 VPS
**Supplementary Video 7**	csLFM realizes high-speed volumetric voltage imaging at 150 VPS with reduced background and enhanced spike resolvability in a *Drosophila* brain

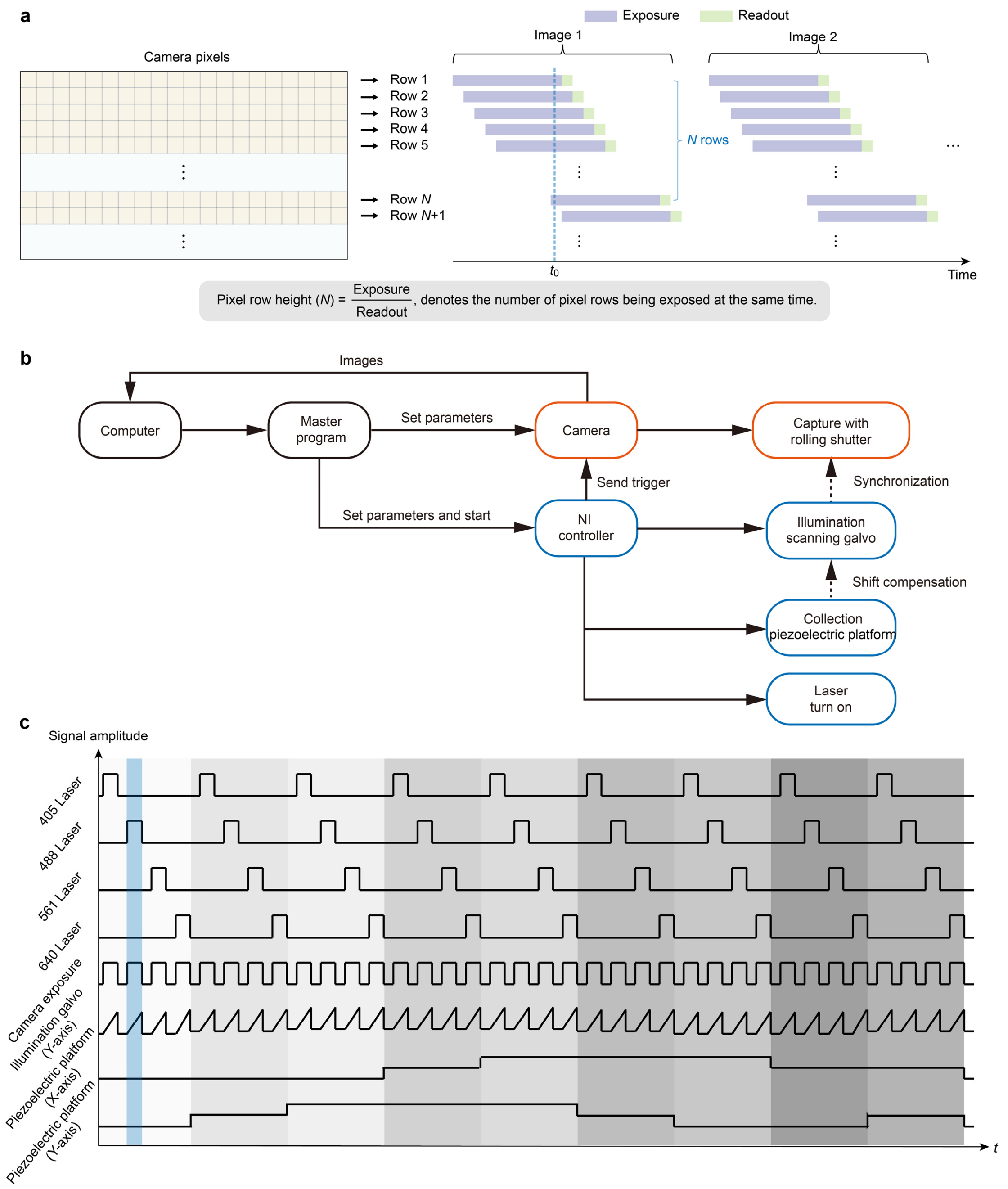

Supplementary Fig. 1 | Illustration of rolling shutter mechanism and csLFM synchronization. **a**, Principle of camera rolling shutter. The camera pixels are exposed and readout row by row with some overlap. The rolling shutter serves as a flexibly variable slit. The ratio of single-row exposure time to interval between adjacent rows can be adjusted, defined as pixel row height, to balance imaging speed and background removal capability of csLFM. Pixel row height reflects the number of pixel rows being exposed at the same time, which also can be considered as confocality degree. For example, the smaller row height allows more background removal but shallower depth of field. **b**, Block diagram of the synchronous control. **c**, Signal waveforms to the synchronized devices. Four channels of lasers are switched on one by one at a high speed for sample excitation. During one image acquisition, the rolling window is synchronized with an axially elongated line-confocal illumination scanned from top row of the field of view to the last row, as shown in the blue shade.

After light-field images of all channels are collected, the piezoelectric tip and tilt platform in the detection path will move to the next position. 3 × 3 scanning mode is illustrated in this schematic, in which different level of grey shades indicate the different 2D scanning positions.

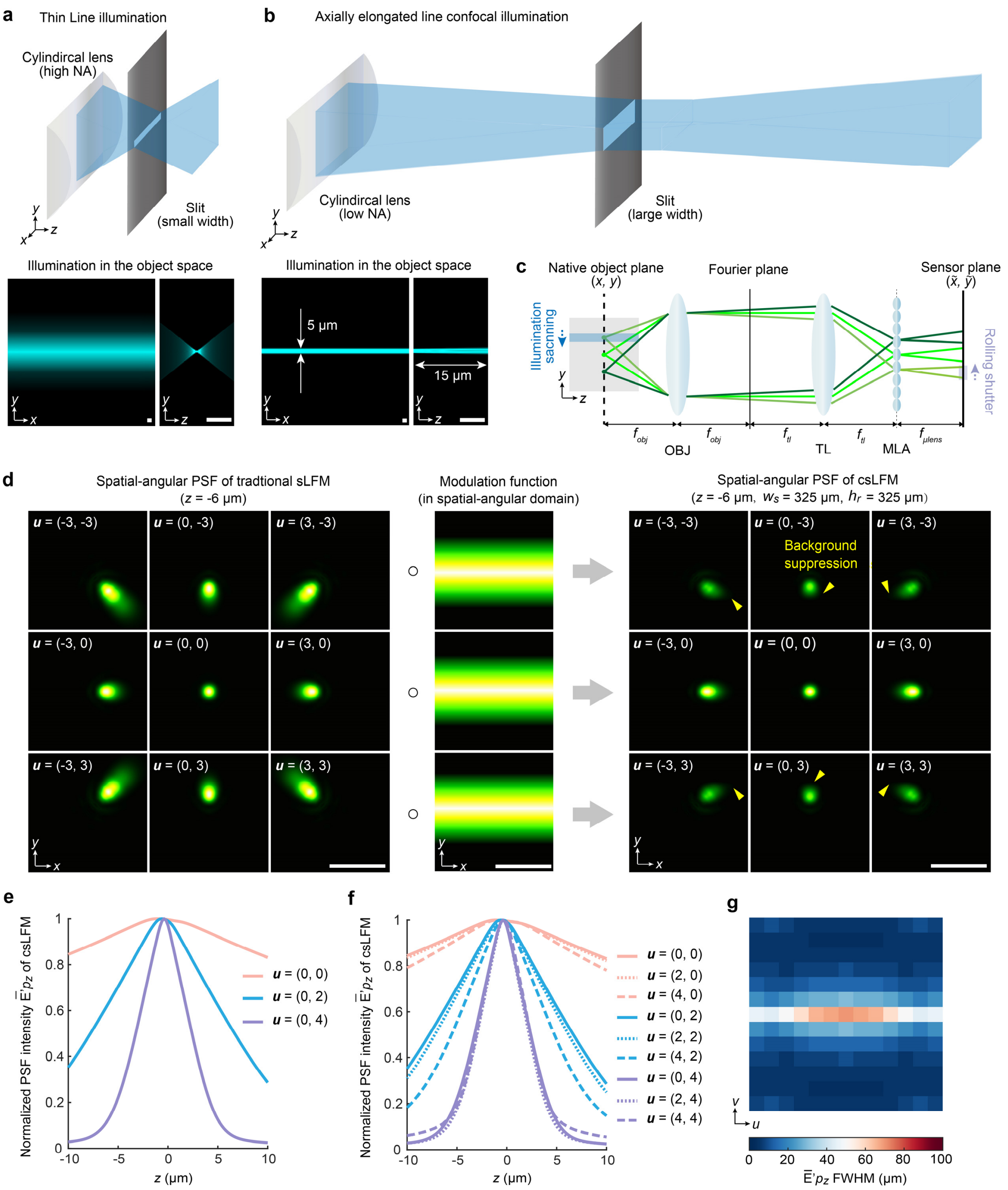

Supplementary Fig. 2 | Illustrations of axially elongated line-confocal illumination and spatial-angular PSF modulation in csLFM. a-b, Comparison of thin line illumination and axially elongated line-confocal illumination. The light is modulated into a line shape by a cylindrical lens, then passes through an optical slit with fixed width. If the cylindrical lens has a small NA coupled with a wider slit, the thin line illumination can be converted into an axially elongated line-confocal illumination. The patterns of thin line illumination and the axially elongated line-confocal illumination in the object space are displayed with orthogonal average intensity projections (AIPs) at the bottom. **c**, The microimaging optical diagram of csLFM. While the illumination is scanned through the object space along the *y* axis, the rolling shutter is sliding at the sensor plane correspondingly to attain a confocal effect. **d**, The effect of confocal modulation that converts PSF of sLFM into that of csLFM with yellow arrows indicating the background suppression by csLFM.

The csLFM PSF is derived based on the slit size of 11 AU. **e-f**, The energy curves of csLFM PSF versus axial positions, spanning different angular dimensions of *u* (**e**) and *v* (**f**). The normalized PSF energy $\overline{E}'_{p_z}$ is defined in Methods section. **g,** The heat map of the FWHMs of the $\overline{E}'_{p_z}$ alone the *z* axis. Scale bars, 5 μm (**a**) and 10 μm (**d**).

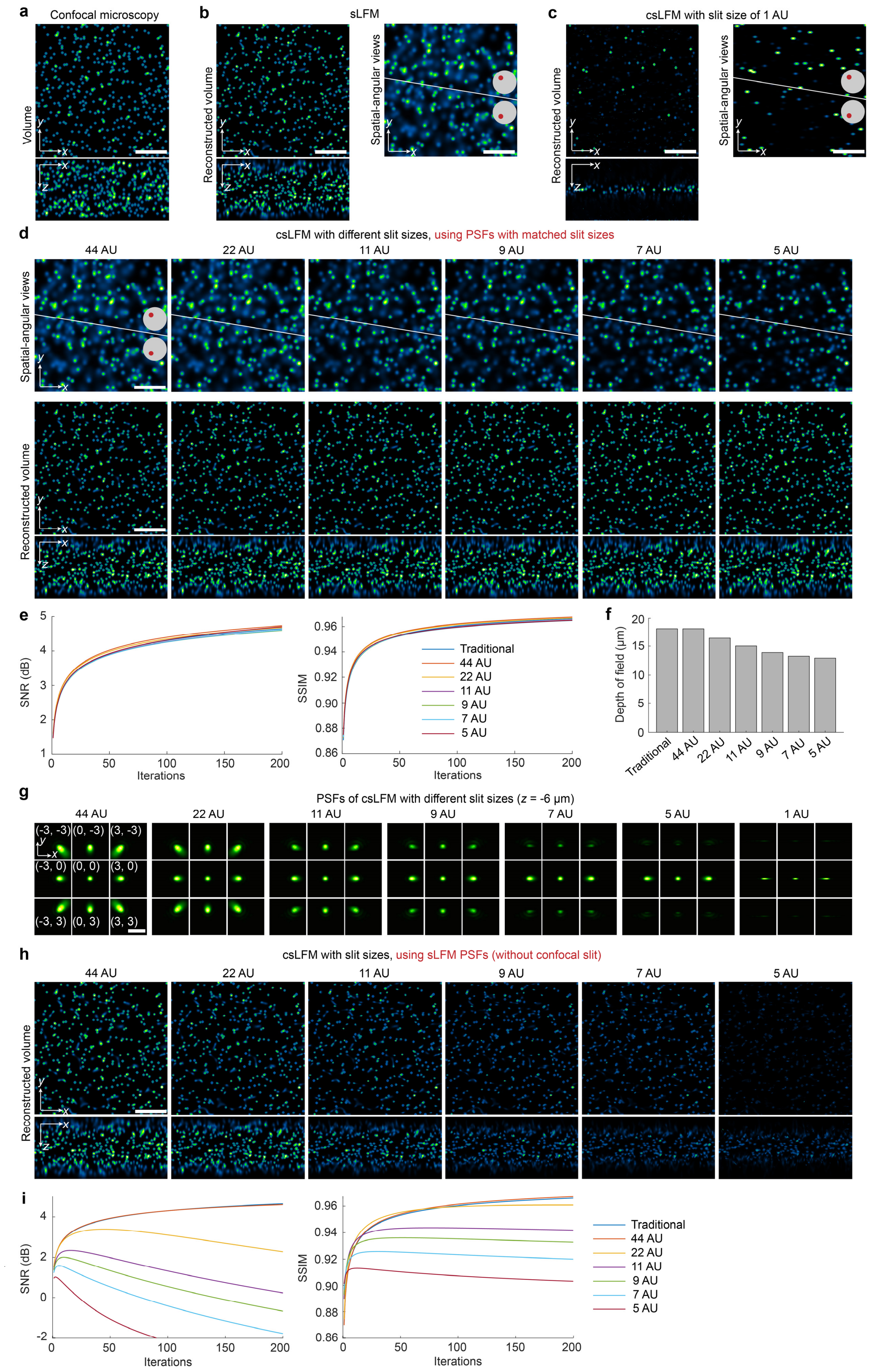

Supplementary Fig. 3 | Numerical analysis of the influence of different slit sizes on axial coverage and reconstruction performance. a, Orthogonal averaged intensity projections (AIPs)

of simulated densely distributed 1-μm-diameter beads by confocal microscopy. The volume is obtained with axial scanning to get the volumetric imaging. **b**, Orthogonal AIPs and corresponding angular views by sLFM. Without background, sLFM results can also be regarded as reference, but with slightly shallower DOF than scanning confocal ones. **c**, Results of csLFM with small slit size, showing a very shallow DOF. **d**, Results of csLFM with different slit sizes. **e**, Convergence curves of the SNR and SSIM indices versus iteration numbers with different parameters applied to csLFM. **f**, The depth of fields (DOFs) of sLFM and csLFM with different slit sizes. The DOF is denoted as the axial range of resolution decreasing by half. When slit size decreases, csLFM achieves similar performances in SNR and SSIM with a very slight degradation in axial coverage. All images were captured with a 63×/1.4 NA oil-immersion objective. **g**, The lateral PSF of different angular PSF measurements of csLFM with different slit sizes at z = -6 μm. **h-i**, The same reconstructions and evaluations as demonstrated in **d-e**, but proceeded with PSFs of sLFM without considering the excitation slit, suggesting that incorrect PSF model would lead to poor performance. Each sub-image in **a-d** and **g** is displayed in scales normalized to the minimum and maximum intensities of sLFM results in **b**. Except for special notes, PSFs used for reconstruction are all correspondingly matched to the parameters of csLFM. Scale bars, 10 μm (**a-d**, **g-h**).

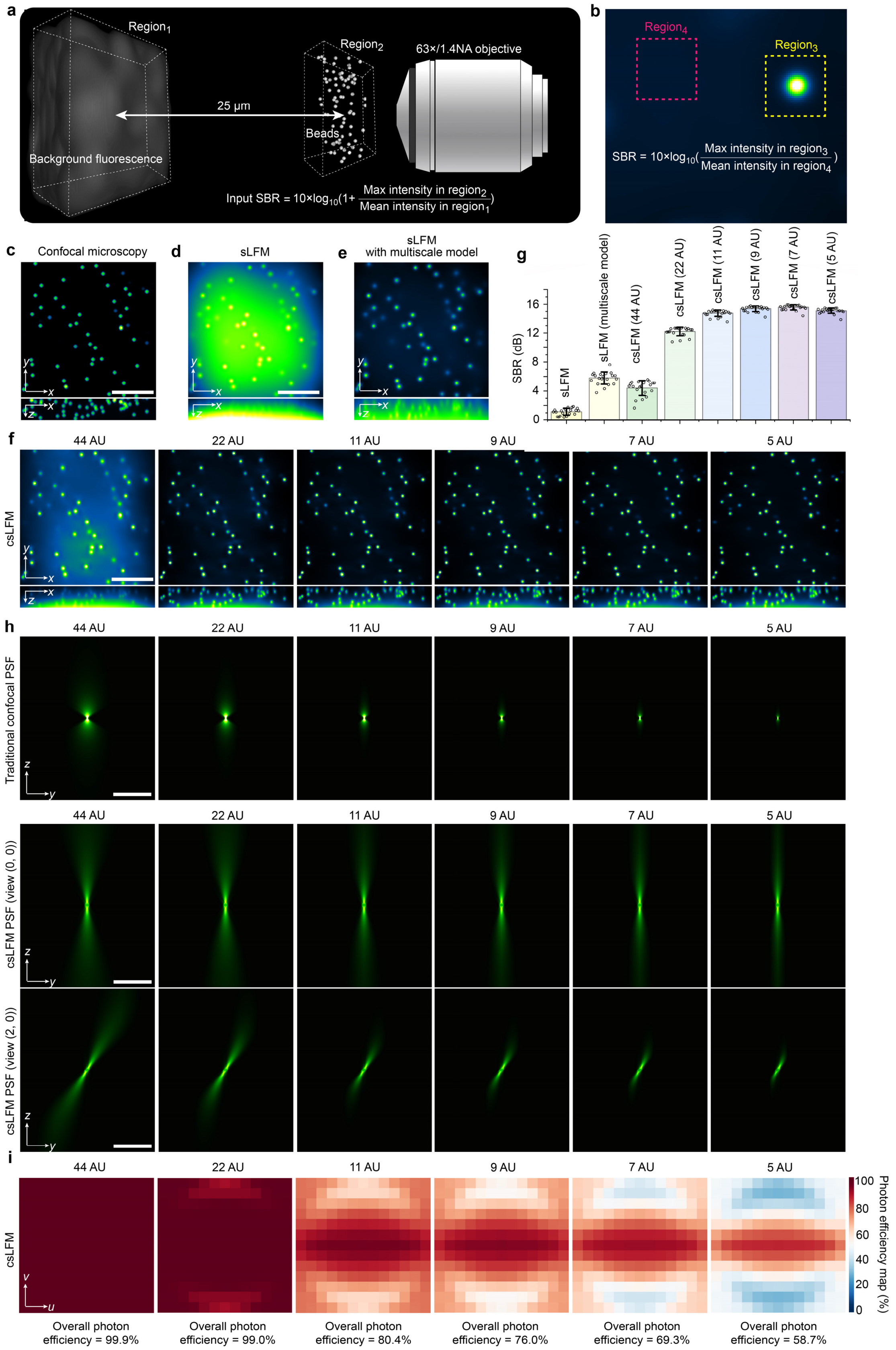

Supplementary Fig. 4 | Numerical analysis of the influence of different slit sizes on SBR and photon efficiency. a, Schematic of the subsequent simulation experiments. 1-μm-diameter fluorescent beads were randomly distributed in the range of 8 microns above and below the native

image plane. Background fluorescence was derived from the summation of multiple Gaussian-distributed intensities, which was at the distance of 25 microns from the native image plane. Both the background and the beads were imaged under a 63×/1.4 NA oil-immersion objective. The background level can be reflected by the input SBR. **b**, The SBR calculation. The SBR is defined by the tenfold logarithmic ratio between signal (maximum intensity of selected ROIs) and background (average intensity of regions without beads). **c-e**, Orthogonal averaged intensity projections (AIPs) by confocal microscopy, sLFM, sLFM with multiscale model, with the input SBR of ~2 dB. **f**, Orthogonal AIPs by csLFM with different slit sizes. When slit size decreases, csLFM achieves better optical sectioning for background rejection. **g**, Bar graphs of SBR achieved by sLFM, sLFM with multiscale model and csLFM with different slit sizes. When slit size is less than 11 AU, the characterized SBR tends to converge. 20 typical beads in each group were selected for SBR calculations. Data are represented as means ± STDs. **h**, Comparisons of the *yz* planes of the PSFs for traditional confocal microscopy and csLFM with different slit sizes. **i**, The photon efficiency maps and overall values of csLFM with different slit sizes. The photon efficiency is calculated as the ratio of csLFM energy (the sum of intensities measured by csLFM) to sLFM energy (the sum of intensities measured by sLFM) for each angular view within 6-μm axial coverage. The photon efficiency of sLFM is considered as 100%, since sLFM collects all emitted light without any blocking. Each sub-image in **c-f** is displayed in scales normalized to its own minimum and maximum intensities. Scale bars, 10 μm (**c-h**).

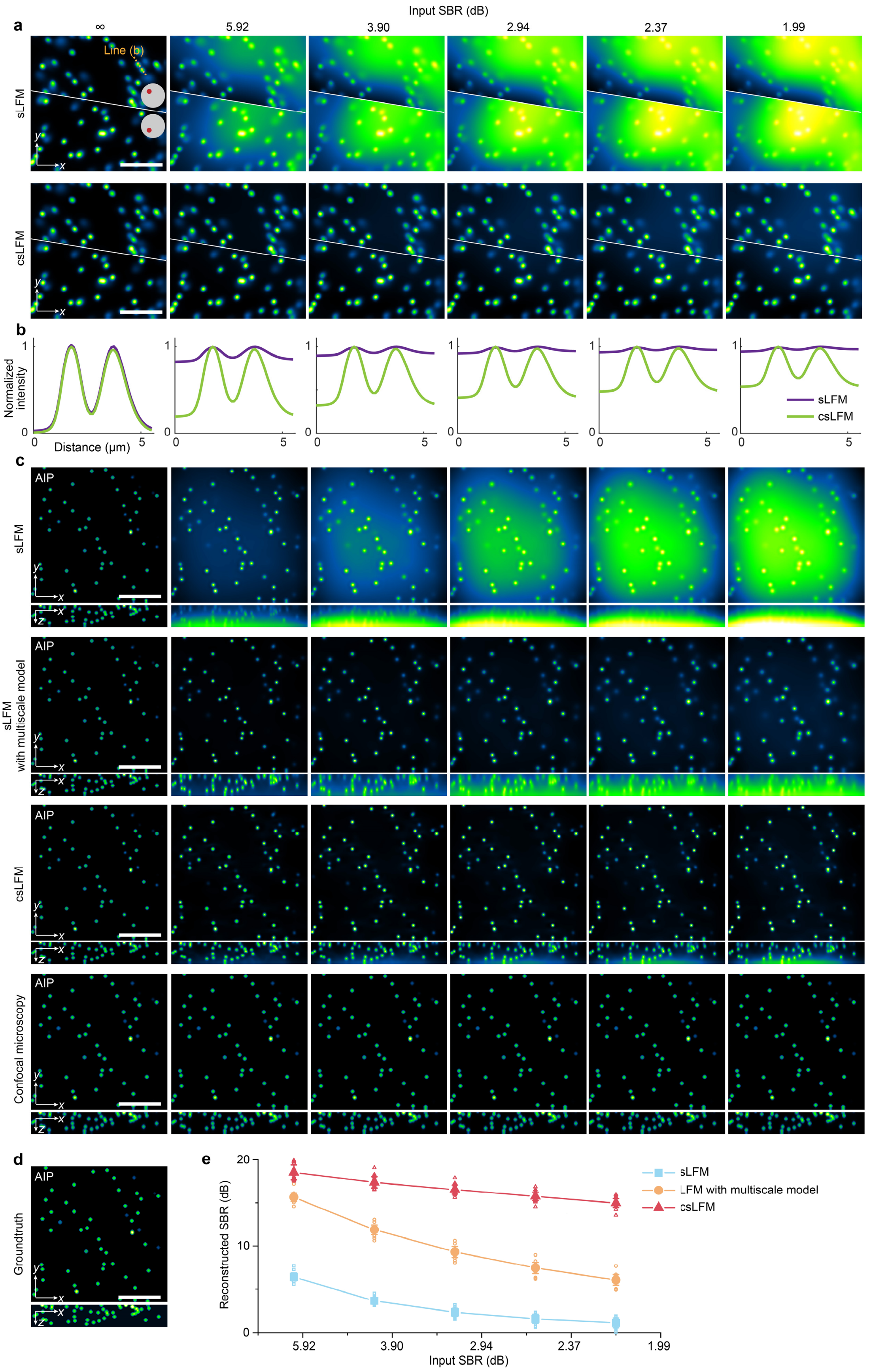

Supplementary Fig. 5 | Numerical analysis of the imaging performance of sLFM and csLFM with increasing background levels. a, The spatial-angular measurements by sLFM and csLFM

with increasing background levels. Two example angular views are displayed. **b**, Comparison of normalized intensity profiles of orange lines across adjacent beads marked in **a**. csLFM significantly improves image contrast, while sLFM results show almost no contrast when input SBR is reduced to ~2 dB. **c**, Corresponding orthogonal averaged intensity projections (AIPs) of reconstruction results, by sLFM, sLFM with multiscale model and csLFM, respectively. Different columns show results at different input SBR. Compared to multiscale LFM, csLFM reduces the influence of background fluorescence more significantly. Point-scanning confocal results are shown in the last row as reference. **d**, Ground truth. **e**, Reconstructed SBR curves of beads at different background levels. 20 typical beads in each group were selected for SBR calculations. Data are represented as means ± STDs. Each sub-image is displayed in scales normalized to its own minimum and maximum intensities. Scale bars, 10 μm (**a**, **c**-**d**).

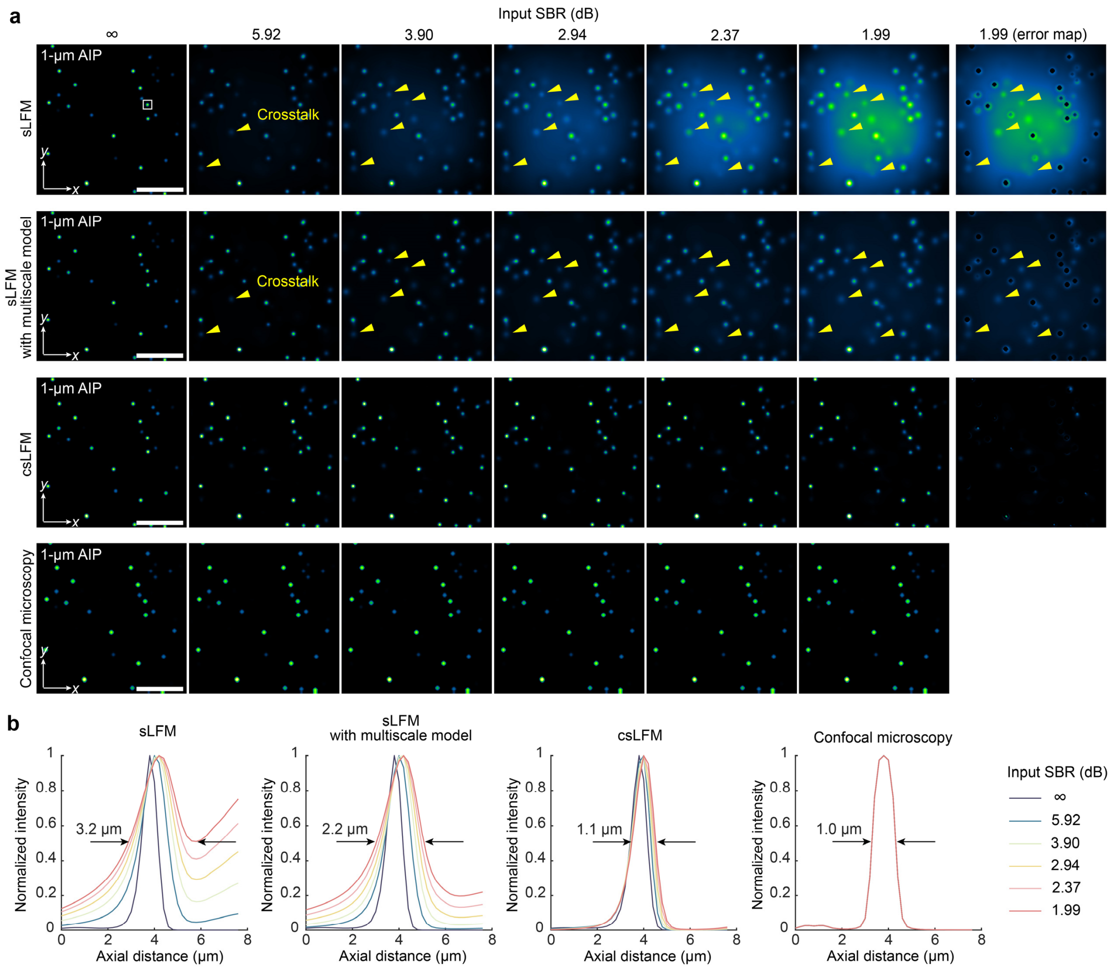

Supplementary Fig. 6 | Numerical analysis of optical sectioning capability and axial performance of sLFM and csLFM with increasing background levels. a, The same reconstructed results as those in Supplementary Fig. 5c, but displayed with AIPs across 1-μm axial range near the native image plane. The rightmost column shows the error maps of sLFM, sLFM with multiscale model and csLFM. Error maps were calculated by subtracting the confocal image from corresponding images in every row. There exists signal crosstalk from other depths in the results of sLFM and sLFM with multiscale model, as indicated by yellow arrows. On the contrary, csLFM exhibits desirable optical sectioning capability that keeps high consistency with confocal microscopy. Each sub-image is displayed in scales normalized to its own minimum and maximum intensities, except for error maps. **b**, Normalized axial intensity profiles along the bead marked by white box in **a**, for different methods and background levels. Arrows indicate the estimated axial resolutions. Scale bars, 10 μm.

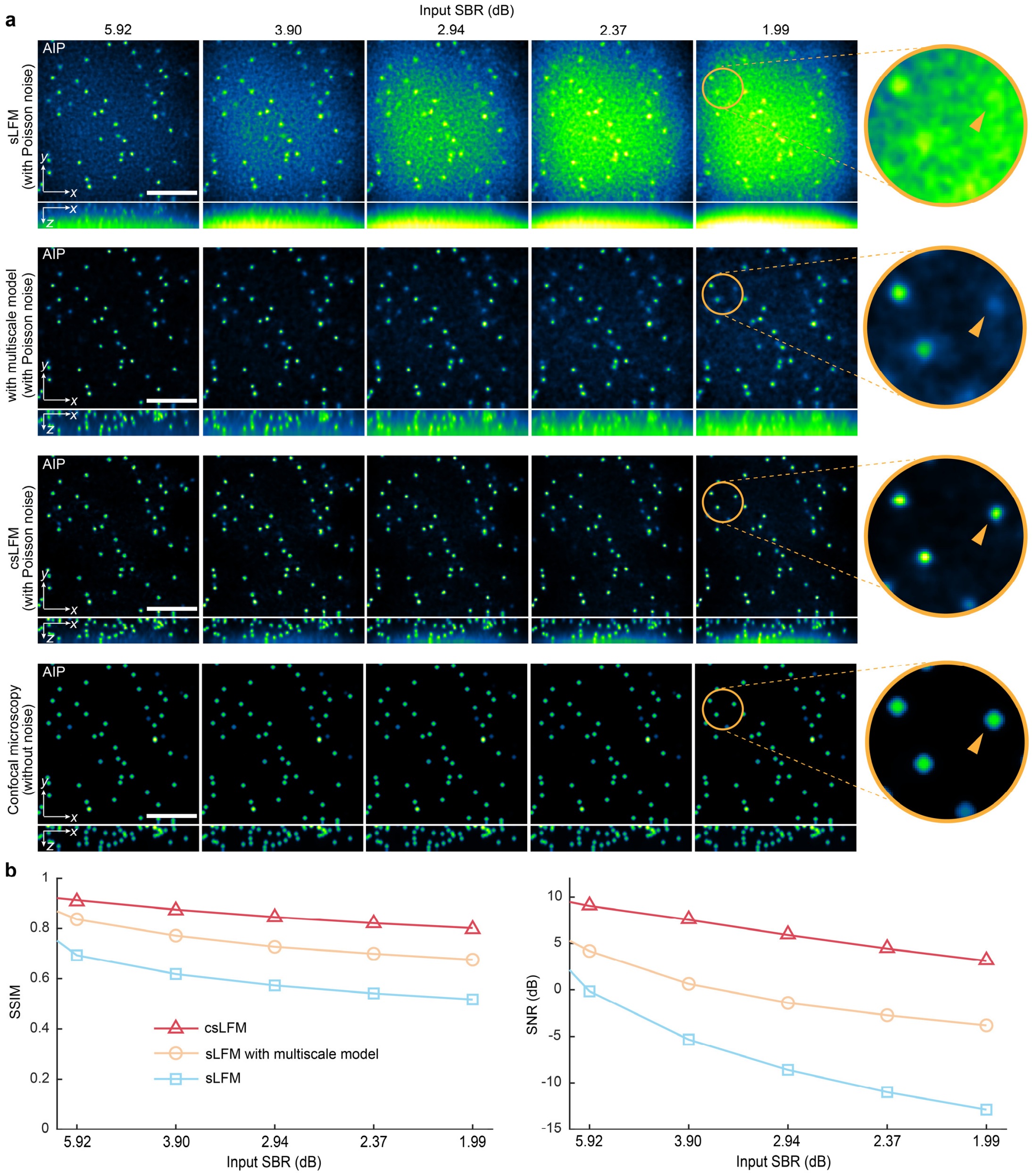

Supplementary Fig. 7 | Numerical analysis of the influence of shot noise in csLFM with increasing background levels. a, Orthogonal averaged intensity projections (AIPs) by sLFM, sLFM with multiscale model and csLFM, at the existence of mixed Poisson-Gaussian noise, which is correlated with the intensity of measurements. The bit depth captured by the detector was set to 16 and the relative photon number of the maximum intensity (65,535) was set to 60. Content-independent Gaussian noise was assumed relatively weak with its variance of 100, which keeps the Poisson component dominant. Confocal results without noise are displayed in the last row as reference. The rightmost column shows the enlarged regions, revealing that beads contaminated by severe shot noise can be clearly distinguished by csLFM, as indicated by orange arrows. Each sub-image is displayed in scales normalized to its own minimum and maximum intensities, except for enlarged views. **b**, SSIM and SNR curves at different background levels applied for different methods. Scale bars, 10 μm (**a**).

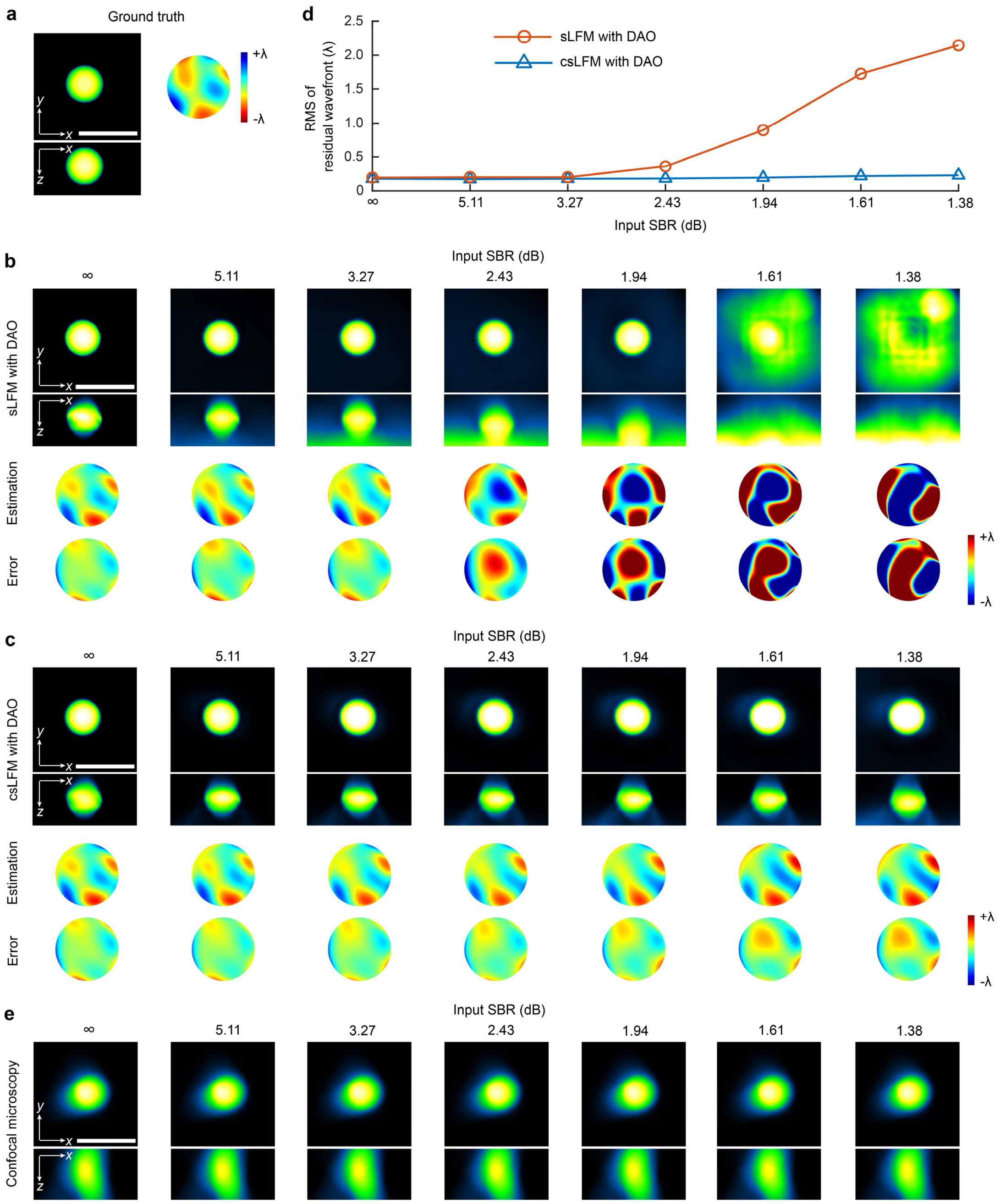

Supplementary Fig. 8 | Numerical analysis of digital adaptive optics (DAO) accuracy in csLFM with increasing background levels. a, Orthogonal averaged intensity projections (AIPs) of a simulated 6-μm spherical bead and an artificially synthetic wavefront, used as ground truth. **b-c**, Upper, Orthogonal averaged intensity projections (AIPs) by sLFM with DAO and csLFM with DAO. Lower, estimated aberration wavefronts by DAO and corresponding error maps. **d**, Curves of residual wavefront errors versus different background levels, obtained by sLFM with DAO and csLFM with DAO. **e**, Confocal results under the aberration. Each sub-image is displayed in scales normalized to its own minimum and maximum intensities. Scale bars, 10 μm (**a-c**, **e**).

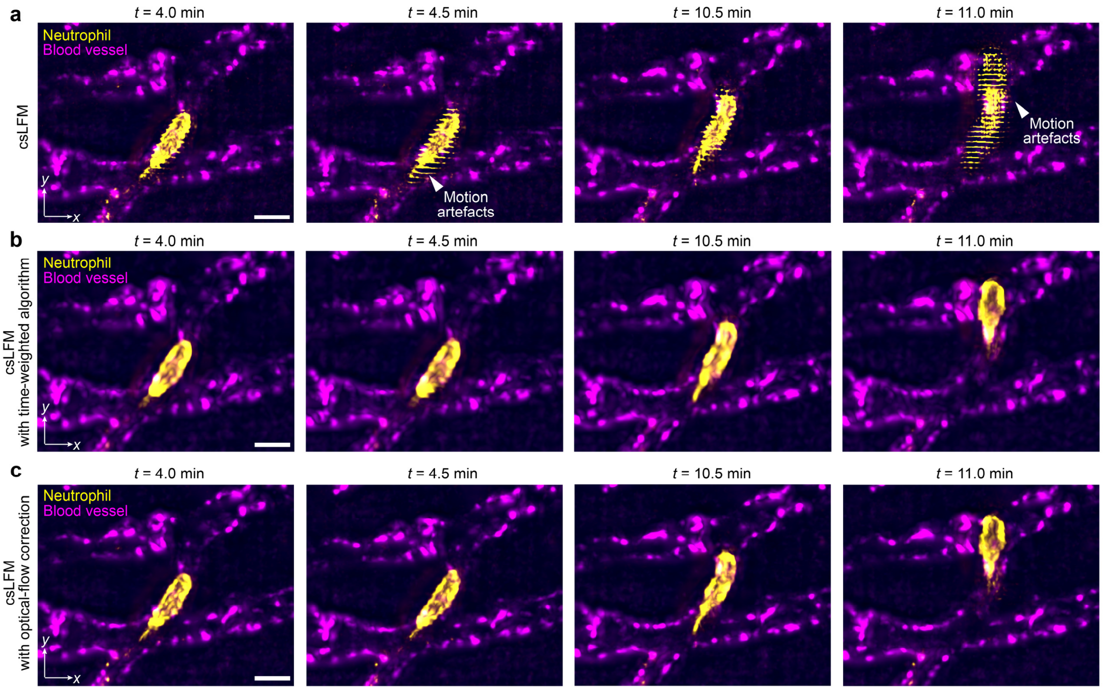

Supplementary Fig. 9 | Motion artefact correction for csLFM imaging. a-c, Maximum intensity projections (MIPs) of Ly6G-labelled neutrophils flowing in the vessels of mouse livers at different time frames, obtained by csLFM without any motion correction (**a**), csLFM with time-weighted algorithm (**b**) and csLFM with optical-flow-based correction (**c**). Arrow indicates motion artefacts. Scale bars, 10 μm.

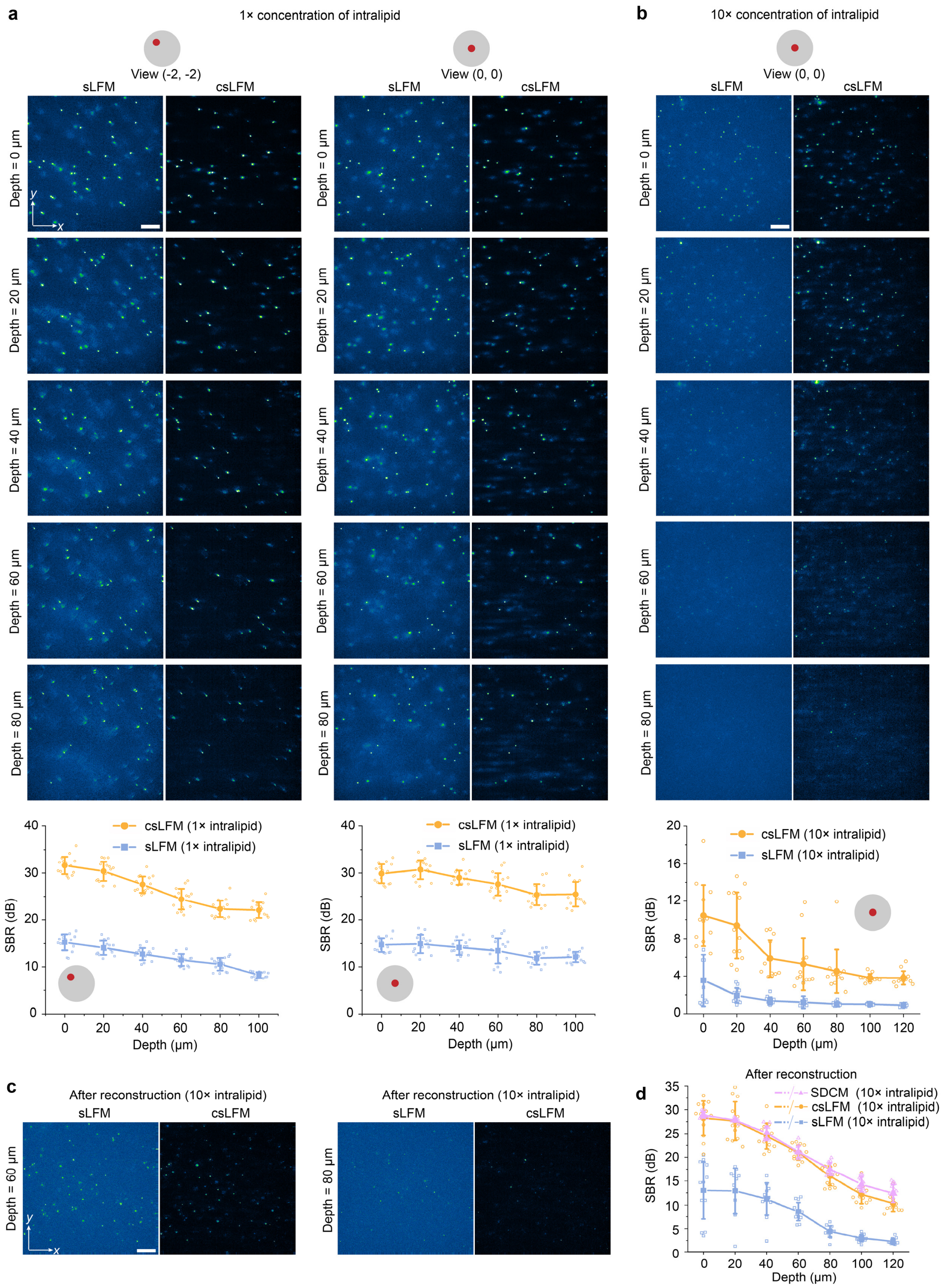

Supplementary Fig. 10 | Experimental SBR characterization in different angular measurements of intralipid-based mixture. a, Example measured views of sLFM and csLFM using a 63×/1.4NA oil immersion objective lens at different penetration depths for comparisons. csLFM shows significantly enhanced image contrast over sLFM. Attached below are SBR curves versus different penetration depths obtained by sLFM and csLFM. Relative to sLFM, csLFM achieves a remarkable SBR improvement of over 12 dB at almost all depths. Angular components away from the center angle along the rolling direction, exhibit higher SBR improvement. Each

center-view image is displayed after subtracting the camera bias with a grayscale of 100. **b**, Center-view measurements and SBR characterization using the mixture with 10× higher intralipid concentration than that in **a**. **c**, Maximum intensity projections (MIPs) of the reconstructions at representative depths of 60 μm and 80 μm, obtained by sLFM and csLFM. **d**, Curves of SBR versus different penetration depths in the same concentration of intralipid for sLFM, csLFM and SDCM. Data are represented as mean ± standard deviation (STD). The data of sLFM and csLFM was captured with a 63×/1.4NA oil-immersion objective lens, while the SDCM data was captured with a 40×/1.3NA oil-immersion objective lens. 12 typical beads for each block covering about 20 μm depth range were selected for SBR calculation. It is noted that the curves in **d** are also displayed in Fig. 2c. Scale bars, 30 μm (**a**-**c**).

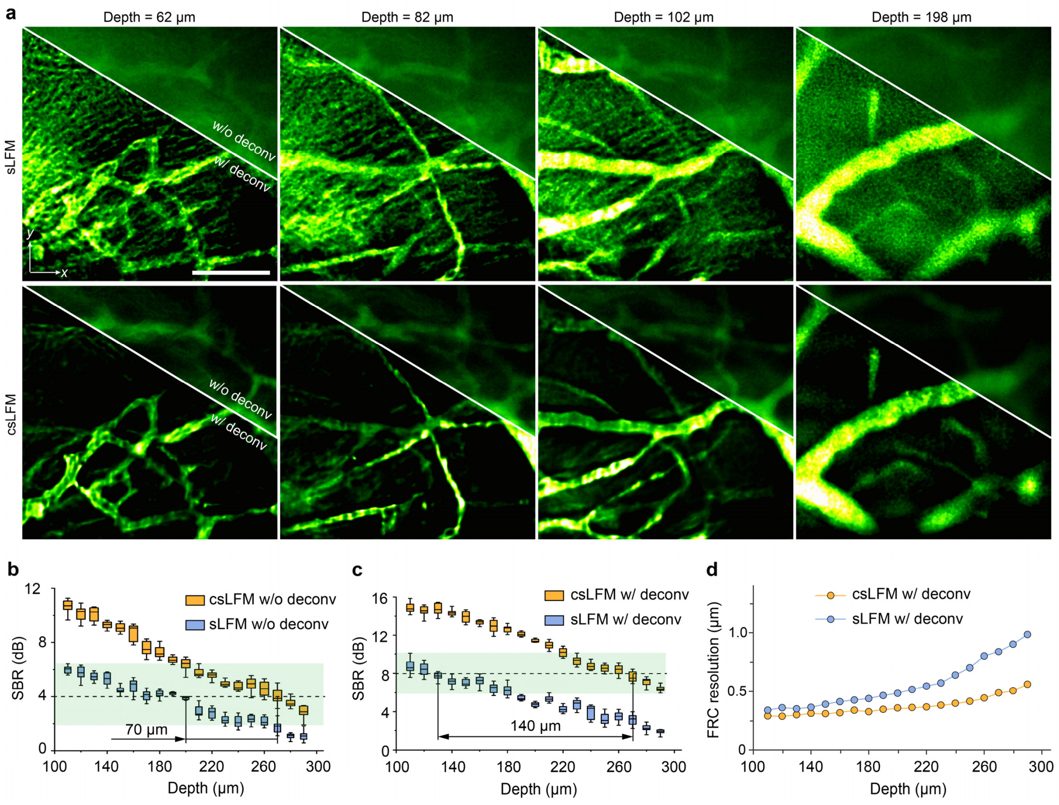

Supplementary Fig. 11 | Experimental evaluation of SBR and resolution with different penetration depths in a mouse brain *in vivo*. **a**, Representative *xy*-slices acquired by sLFM (upper) and csLFM (lower) of vascular structures labelled with AF647 dye in a mouse brain *in vivo*. Images before and after 3D deconvolution are displayed. **b-c**, Boxplot showing SBR of sLFM and csLFM at different imaging depths. The results with (**b**) and without (**c**) 3D deconvolution are displayed. To quantify the SBR improvement of csLFM, we manually segmented the image into small regions, each occupying 25 × 25 μm^2 and including at most one blood vessel, in order that SBR could be calculated as described in Methods section. The green shallow in each panel is the range of values that both yellow and blue curves can cover. The dashed line is the median of green shallow, indicating the improvement of imaging depths with the same SBR. The boxplot format: center line, median; box limits, lower and upper quartiles; whiskers, 0th–100th percentiles excluding outliers. n = 12 vascular regions at each depth were selected for SBR calculation. **d**, Resolution curves at different imaging depth by csLFM and sLFM. The resolutions were estimated by the Fourier ring correlation (FRC) analysis. Scale bars, 100 μm (**a**).

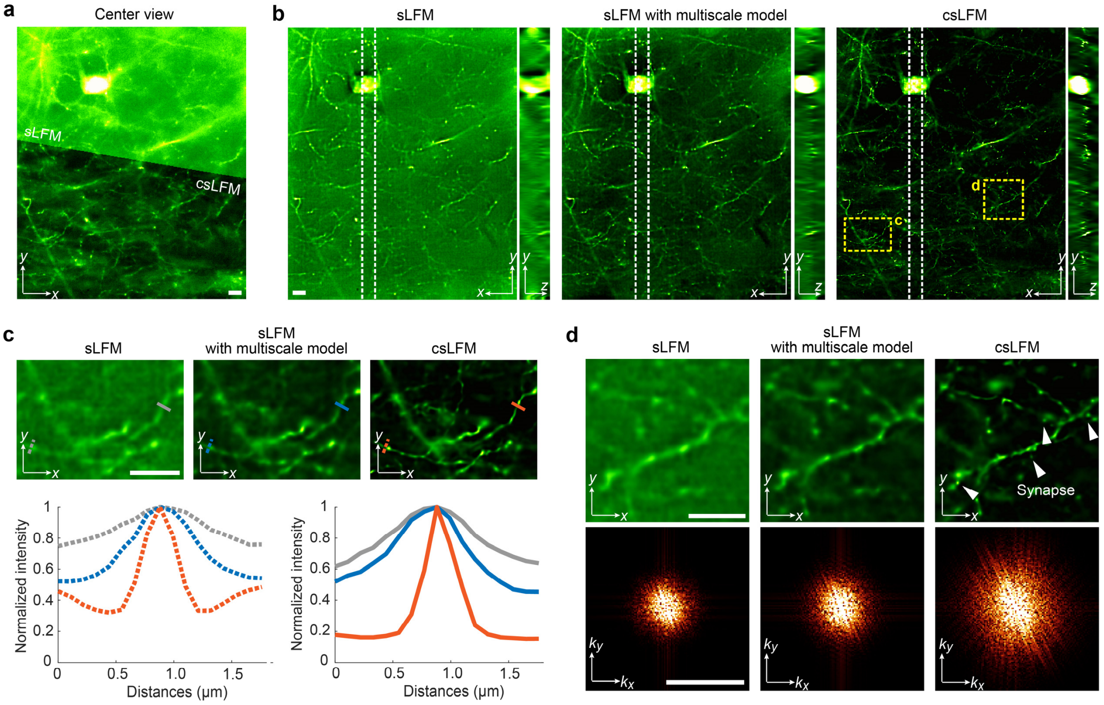

Supplementary Fig. 12 | Experimental comparisons of a 300-μm-thick Thy1-YFP mouse brain slice imaged by sLFM, sLFM with multiscale model and csLFM. a, Center-view measurements by sLFM (top) and csLFM (bottom), both of which were captured with a 63×/1.4NA oil immersion objective. The imaging depth is approximately 100 microns above the bottommost structures. **b**, Orthogonal averaged intensity projections (AIPs) by sLFM, sLFM with multiscale model and csLFM. The white dashed lines indicate the regions for *yz* projection. **c**, Enlarged views marked in **b**. Normalized intensity profiles along the marked lines are shown below, exhibiting the visible narrower FWHMs and higher SBR by csLFM. **d**, Enlarged views marked in **b** and corresponding Fourier transforms. Synapse details that are contaminated by strong background and shot noise it induces in the results of sLFM and sLFM with multiscale model, can be resolved by csLFM, as indicated by white arrows. The Fourier spectrums suggest higher resolution of csLFM, which is originated from the suppression of background-induced shot noise and more details to be detected. Scale bars, 10 μm (**a-d**) and 6 μm^{-1} (**d**).

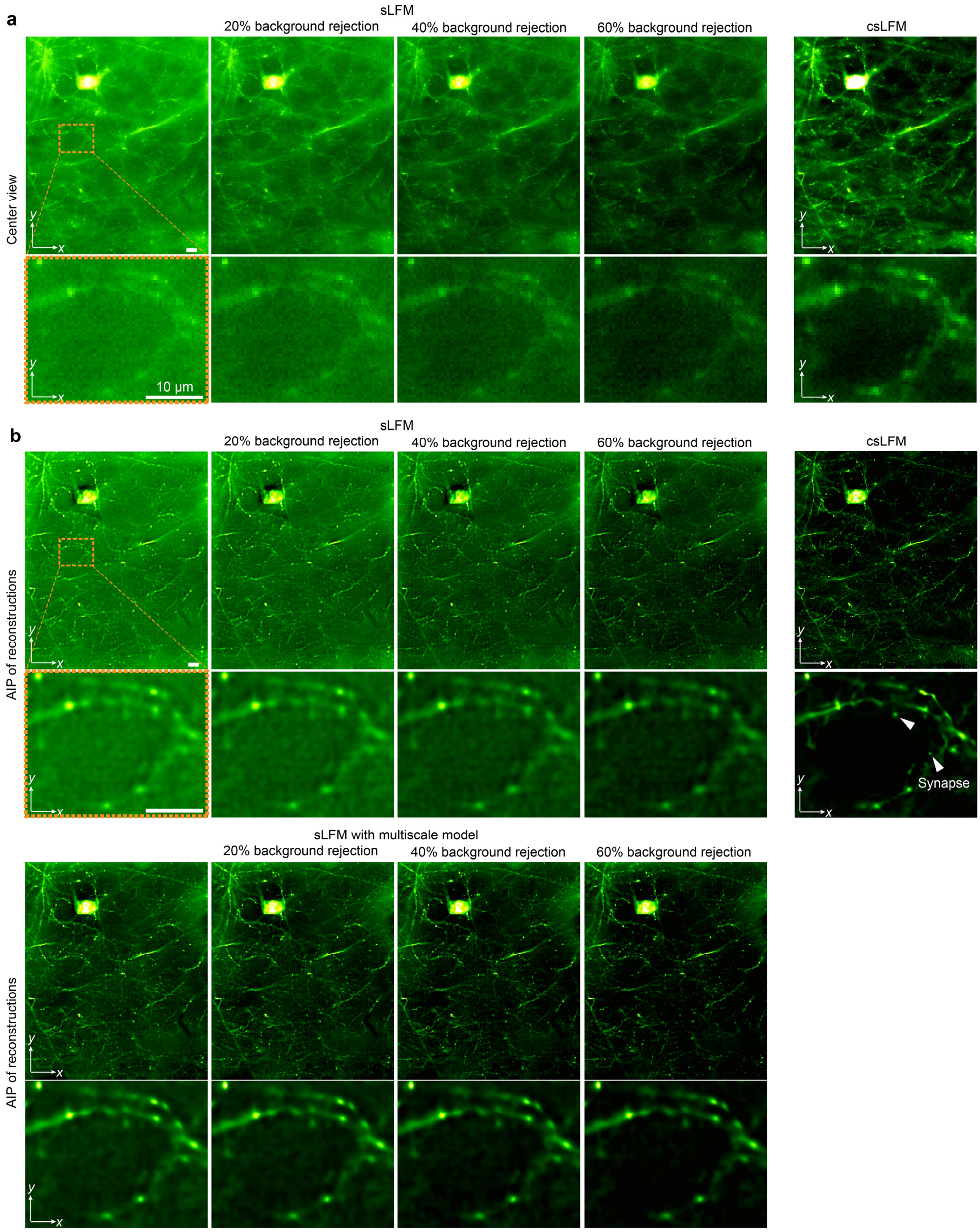

Supplementary Fig. 13 | Experimental analysis of the background-suppressed performance of csLFM and sLFM with direct background subtraction. a, Center views and enlarged regions by csLFM and sLFM with different levels of direct background subtraction. The sample is the same brain slice shown in Supplementary Fig. 12. **b**, Averaged intensity projections (AIPs) and enlarged views after reconstruction with different levels of direct background subtraction. The white arrow points to synapses, which cannot be distinguished by sLFM with simple background removal, even if using the multiscale model. Scale bars, 10 μm.

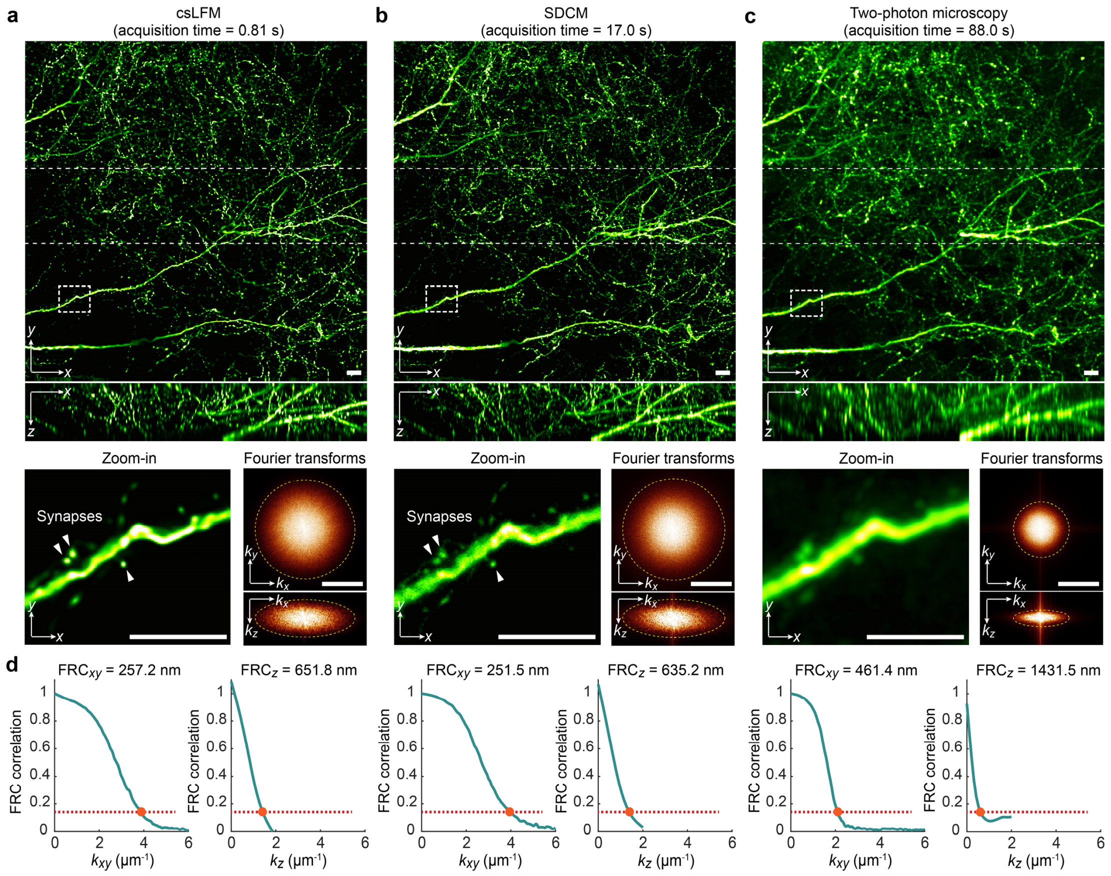

Supplementary Fig. 14 | Experimental comparisons of a 300-μm-thick Thy1-YFP mouse brain slice imaged by csLFM, SDCM and two-photon microscopy. a-c, Orthogonal averaged intensity projections (AIPs) by csLFM (**a**), SDCM (**b**) and two-photon microscopy (**c**) are shown at the top. The white dashed lines indicate the regions for *xz* projection. The data of csLFM was captured with a 25×/1.05 NA water-immersion objective lens, the data of two-photon microscopy was captured with the same 25×/1.05 NA water-immersion objective lens, while SDCM data was captured with a 40×/1.1 NA water-immersion objective lens. Enlarged regions are shown in the bottom left, and Fourier transforms of the 2D orthogonal AIPs are displayed in the bottom right for detailed comparison. Arrows point to the same resolved tiny synapses. **d**, Fourier ring correlation (FRC) curves (green lines) for orthogonal AIPs shown in the top row of panel **a-c**, with the threshold set to 1/7 (dotted red lines). Estimated resolutions by FRC are marked. Scale bars, 10 μm and 3 μm^{-1} (**a-c**).

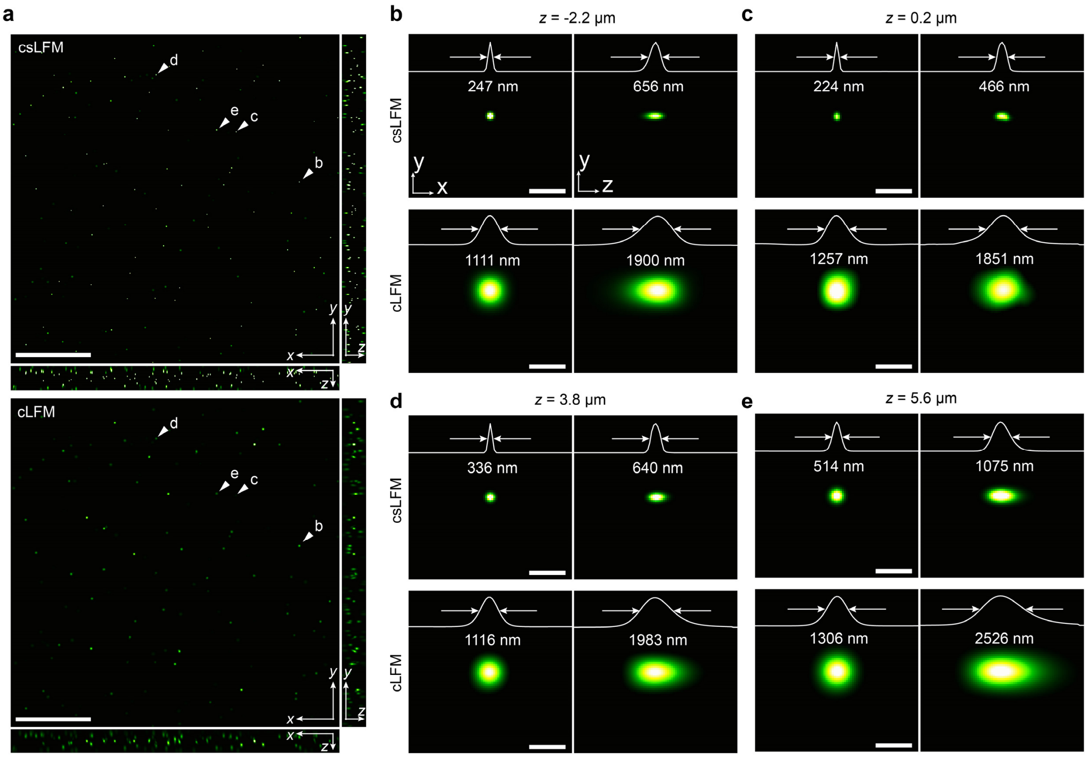

Supplementary Fig. 15 | Resolution characterization of cLFM and csLFM in 3D fluorescence imaging. a, Orthogonal maximum intensity projections (MIPs) of 100-nm-diameter fluorescence beads randomly distributed in 1% agarose obtained by cLFM and csLFM with a 63×/1.4 NA oil-immersion objective. **b-e**, Enlarged regions of different fluorescence beads marked in **a**. Scale bars, 50 μm (**a**) and 2 μm (**b-d**).

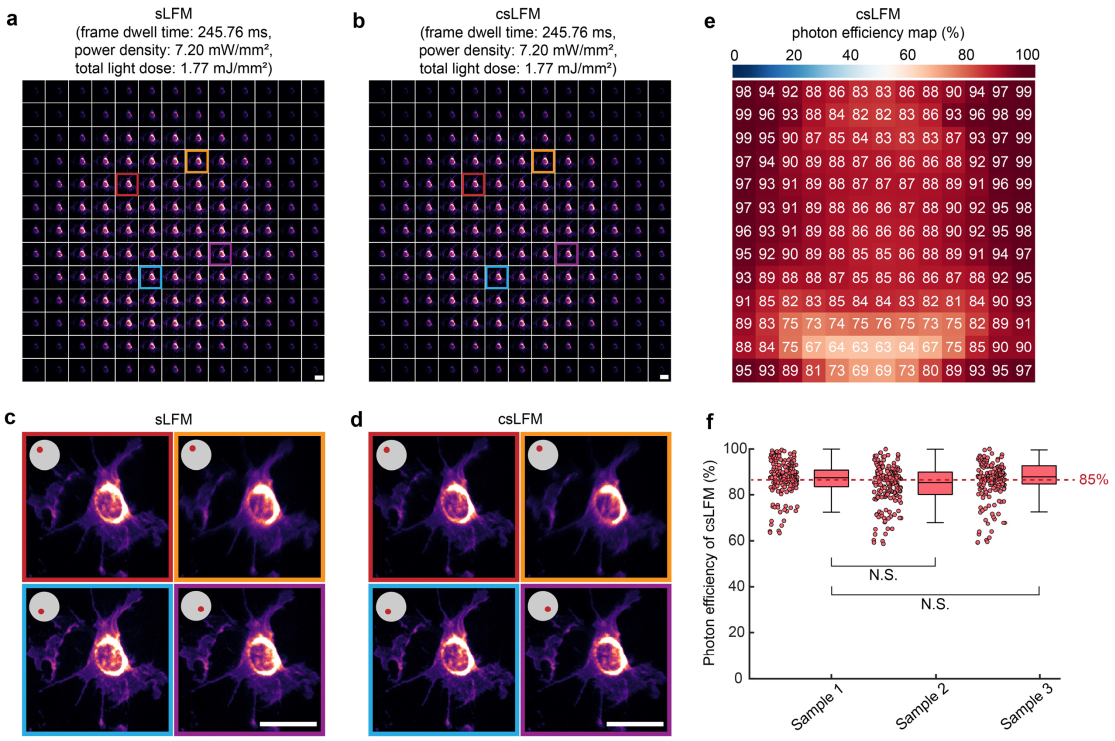

Supplementary Fig. 16 | Photon efficiency analysis of csLFM in imaging thin samples. a-b, 13 × 13 arranged spatial-angular views of a fixed L929 cells with membrane labelling, attached to a glass slide, obtained by sLFM (**a**) and csLFM (**b**), respectively. The laser power density and dwell time during the two acquisitions were set to the same for a fair comparison. **c-d**, Representative views are enlarged. **e**, The photon efficiency map of csLFM. The photon efficiency is calculated as the ratio of csLFM energy (the sum of intensities measured by csLFM) to sLFM energy (the sum of intensities measured by sLFM) for each angular view. The photon efficiency of sLFM is considered as 100%, since sLFM collects all emitted light without any blocking. **f**, Boxplots of the photon efficiencies of csLFM across different angular views and different cells. The average photon efficiency of csLFM is around 85%, as indicated by the dashed line. The boxplot format: center line, median; box limits, lower and upper quartiles; whiskers, 1.5-fold interquartile range. $n = 169$ values for all angular views were plotted. P values were calculated by the two-sided paired t-test. Concretely, $P = 0.076$ for samples 1 and 2, $P = 0.065$ for samples 1 and 3. P value < 0.05 was set as the significantly differential expression. N.S. denotes not significant. Scale bars, 10 μm (**a-d**).

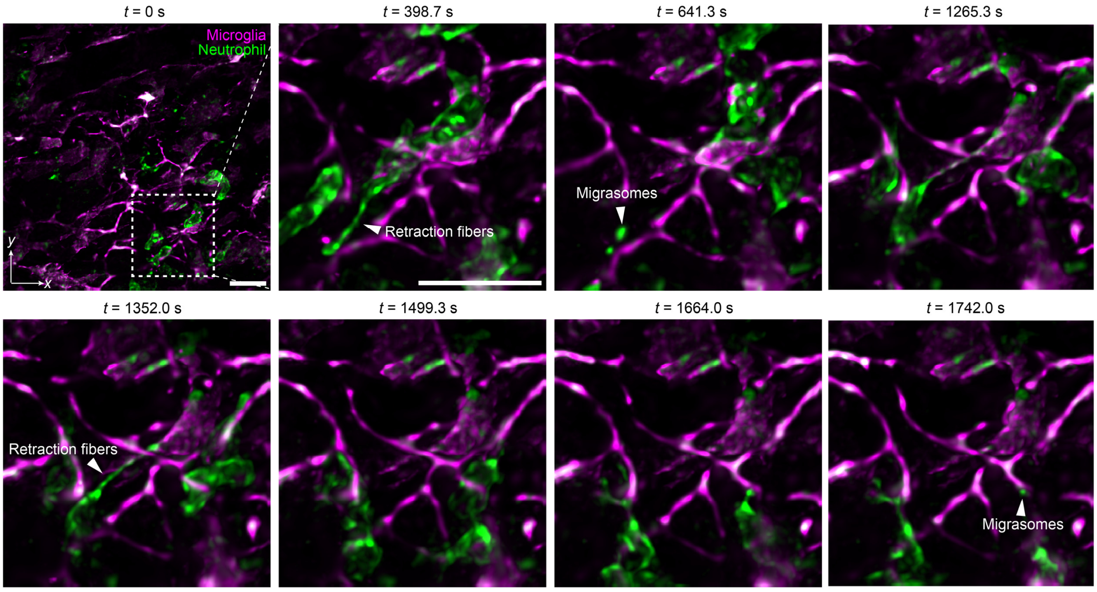

Supplementary Fig. 17 | Formation of retraction fibers and migrasomes in mammals validated by two-photon synthetic aperture microscopy (2pSAM). MIPs and enlarged regions of neutrophils (green) and microglia (magenta) in a mouse brain following traumatic brain injury (TBI) model at different time stamps. With the long-term observation, 2pSAM revealed neutrophils migrated actively and produced migrasomes in neighboring microglia. The white arrows indicate retraction fibers and migrasomes. And a migrasome was retained in microglia at t = 1742.0 s. Scale bars, 30 μm.

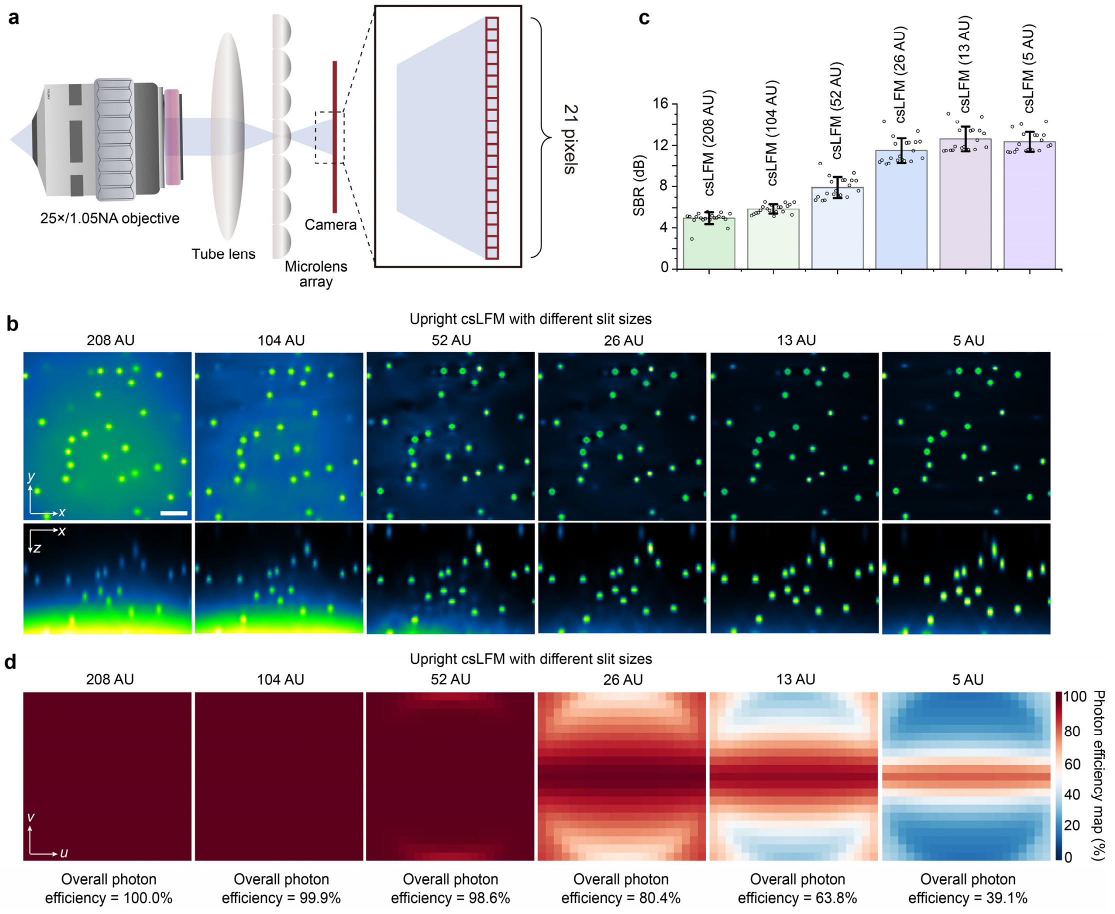

Supplementary Fig. 18 | Numerical analysis of the influence of different slit sizes in the upright csLFM system. a, Experimental setup of upright csLFM system, where a microlens exactly covers 21 × 21 pixels. A 25×/1.05 NA water-immersion objective is applied to cover a larger field of view. The illumination and scanning module are not illustrated for simplicity. **b**, Orthogonal AIPs obtained by csLFM with different slit sizes. Synthetic 3-μm-diameter beads randomly distributed in 3D space were used as the sample, at a relative high background level. **c**, Bar graphs of SBR achieved by csLFM with different slit sizes. When slit size is less than 26 AU, the characterized SBR tends to converge. 20 typical beads in each group were selected for SBR calculations. Data are represented as means ± STDs. **d**, The photon efficiency maps and overall values of csLFM with different slit sizes. The photon efficiency is calculated as the ratio of csLFM energy (the sum of intensities measured by csLFM) to sLFM energy (the sum of intensities measured by sLFM) for each angular view within 25-μm axial coverage. The slit with size of 26 AU enables the upright system to reach the desirable SBR and photon efficiency. Scale bars, 10 μm (**b**).

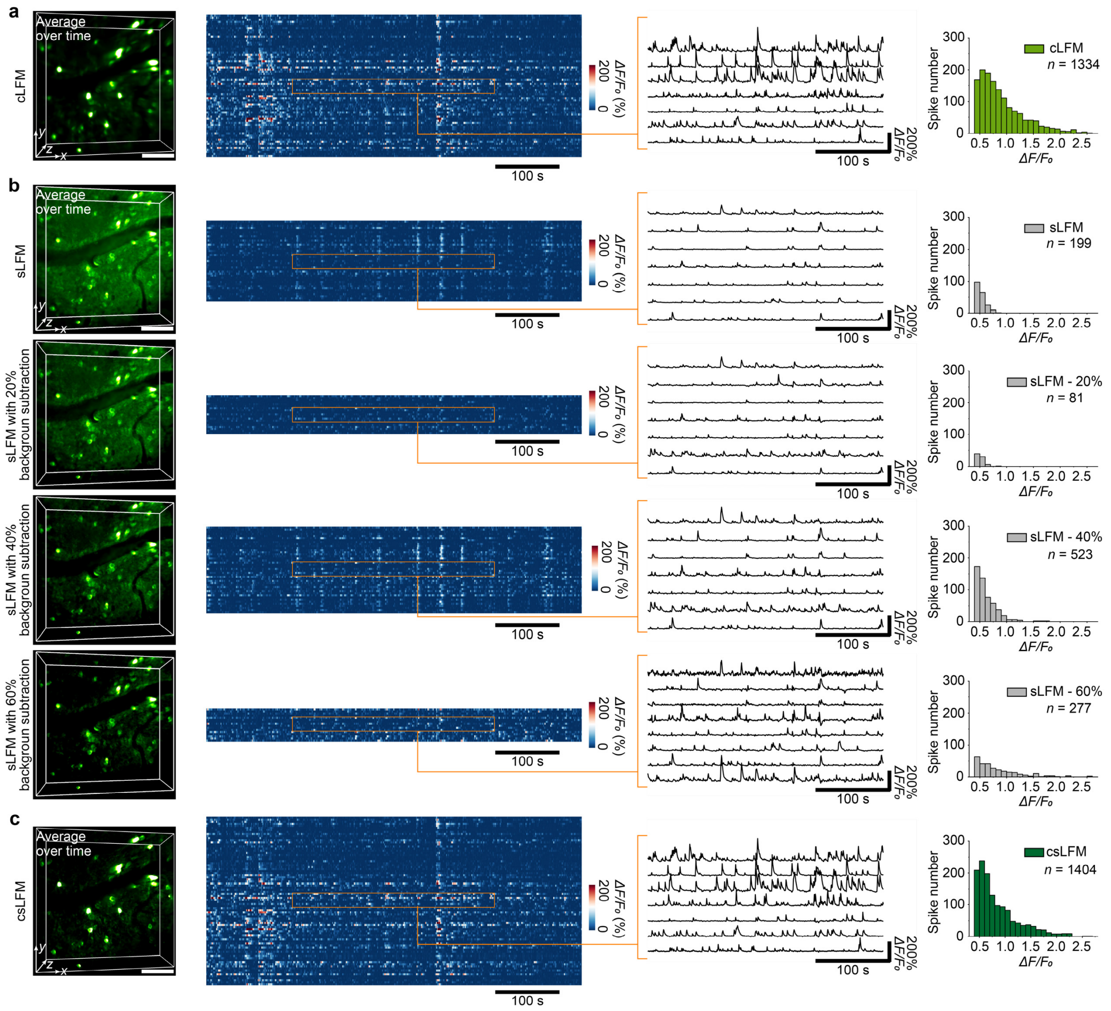

Supplementary Fig. 19 | Detailed comparison of neural imaging in mouse cortex between cLFM, sLFM and csLFM. a-c, Renderings of Fig. 5a (left), corresponding functional $\Delta F/F_0$ traces (middle) and histogram of spike amplitudes (right), obtained from cLFM (**a**), sLFM and its different background subtraction counterparts (**b**), and csLFM (**c**). In the histograms, *n* represents the number of identified spikes. *n* = 1334, 199, 81, 523, 277, 1404 from top to bottom. Scale bars, 100 μm.

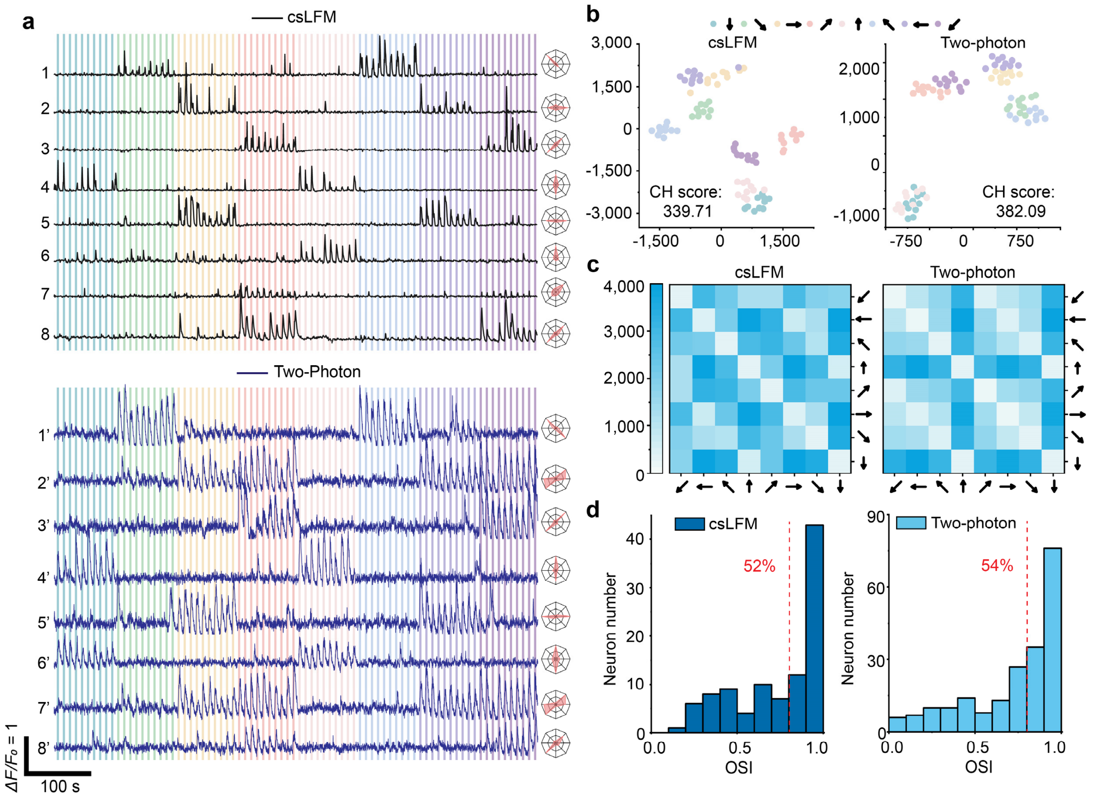

Supplementary Fig. 20 | Comparison of neural recordings under visual stimuli between csLFM and two-photon microscopy. a, Representative temporal traces under the visual stimuli. Eight neurons were analyzed in csLFM and two-photon microscopy. Polar plots for eight directions are also attached to show the tuning curves. **b-d**, Comparisons of dimensionality reduction (t-SNE) maps (**b**), distance matrixes (**c**) and orientation selectivity index (OSI) distributions (**d**) on the extracted temporal traces. Each point in t-SNE maps corresponded to the neural activity for one direction, and the Calinski-Harabasz (CH) score were indicated. n = 106 neurons for csLFM and n = 207 neurons for two-photon microscopy.

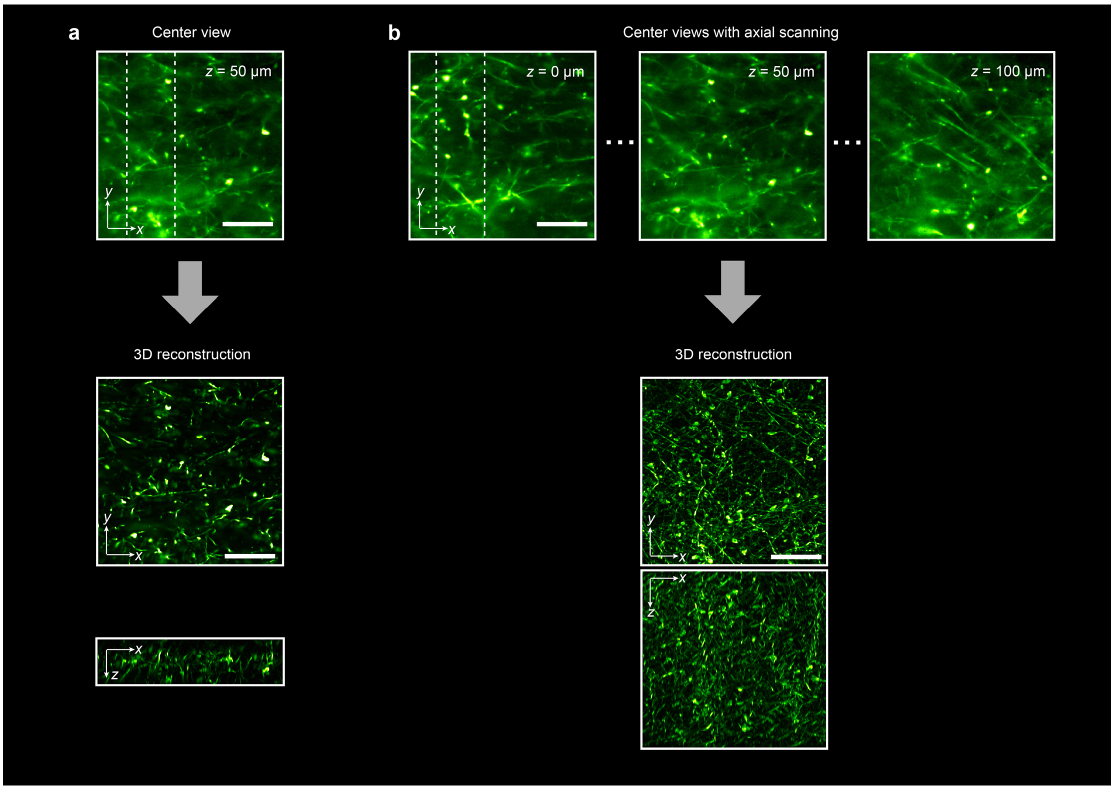

Supplementary Fig. 21 | Extended depth of field of csLFM with axial scanning. a-b, Center views (upper row) and 3D reconstruction renderings (lower row) of a 300-μm-thick Thy1-YFP mouse brain slice, captured by csLFM (**a**) and csLFM with axial scanning (**b**), with a 63×/1.4NA oil immersion objective. The axial scanning was accomplished by a piezo objective scanner, with the axial step of 10 μm and scanning number of 10. With the superior capability of optical sectioning and background removal by csLFM, reconstructed volumes at different axial positions can be spliced seamlessly. A multifold increase in depth of field is obtained. Scale bars, 40 μm (**a-b**).

Supplementary Table 1 | Imaging parameters for all fluorescence experiments

	Sample, (imaging T, °C)	Fluorescent label	Line exposure time (total dwell time in one frame) (# time pts)	λ: Power (mW/mm^2)	Volume rate (VPS)	Objective	System mode, angular resolution, scanning number and z-stacking (if applicable)	Confocality parameters (slit size)
1d, S12-S13, SV1	300-μm-thick brain slice 27 °C	Thy1-YFP	245.76 ms (245.76 ms) 1 pts	488: 1.1	-	63×/1.4NA Oil	sLFM, inverted, 13 × 13, 3 × 3	11 AU
			6 ms (245.76 ms) 1 pts	488: 1.1	-		csLFM, inverted, 13 × 13, 3 × 3	11 AU
2a-2b, S10a	Tissue mimicking phantom 27 °C	Yellow-green fluorescent (505/515)	245.76 ms (245.76 ms) 100 pts	488: 0.8	-	63×/1.4NA Oil	sLFM, inverted, 13 × 13, 3 × 3	500 AU
			6 ms (245.76 ms) 100 pts	488: 0.8	-		csLFM, inverted, 13 × 13, 3 × 3	11 AU
2c, S10b-S10d	Tissue mimicking phantom 27 °C	Yellow-green fluorescent (505/515)	245.76 ms (245.76 ms) 1 pts	488: 0.8	-	63×/1.4NA Oil	sLFM, inverted, 13 × 13, 3 × 3, 120 *z*-steps, 1 μm spacing	500 AU
			6 ms (245.76 ms) 1 pts	488: 0.8	-		csLFM, inverted, 13 × 13, 3 × 3, 120 *z*-steps, 1 μm spacing	11 AU

			-<br>(4970 ms)<br>1 pts	488: 0.8	-	40×/1.3NA Oil	SDCM, inverted, 120 *z*-steps, 1 µm spacing	-
2d, SV3	Living mouse spleen 37 °C	CD11c (dendritic cell), CD8 (T cell)	245.76 ms<br>(245.76 ms)<br>1 pts	561: 1.0<br>640: 1.5	-	63×/1.4NA Oil	sLFM, inverted, 13 × 13, 3 × 3	500 AU
			6 ms<br>(245.76 ms)<br>1 pts	561: 1.0<br>640: 1.5	-		cLFM, inverted, 13 × 13, 1 × 1	11 AU
			6 ms<br>(245.76 ms)<br>1 pts	561: 1.0<br>640: 1.5	-		csLFM, inverted, 13 × 13, 3 × 3	11 AU
2f, S15	Fluorescence beads 27 °C	Yellow-green fluorescent (505/515)	6 ms<br>(245.76 ms)<br>100 pts	488: 10.5	-	63×/1.4NA Oil	cLFM, inverted, 13 × 13, 1 × 1	11 AU
							csLFM, inverted, 13 × 13, 3 × 3	
2h (left panel)	Living mouse spleen 37 °C	NK1.1 (NK cell), F4/80 (macrophage)	-<br>(4970 ms)<br>82 pts	561: 1.0<br>640: 2.8	1/30	40×/1.3NA Oil	SDCM, inverted, 75 *z*-steps, 0.2 µm spacing	-
2h (right panel)	Living mouse spleen 37 °C	NK1.1 (NK cell), F4/80 (macrophage)	6 ms<br>(245.76 ms)<br>631 pts	561: 1.0<br>640: 2.8	1/30	63×/1.4NA Oil	csLFM, inverted, 13 × 13, 3 × 3	11 AU
2j	Living mouse spleen 37 °C	NK1.1 (NK cell)	6 ms<br>(245.76 ms)<br>1608 pts	561: 5.0<br>640: 14.0	1/10	63×/1.4NA Oil	csLFM, inverted, 13 × 13, 3 × 3	11 AU
			-<br>(4970 ms)<br>124 pts	561: 5.0<br>640: 14.0	1/10	40×/1.3NA Oil	SDCM, inverted, 75 *z*-steps, 0.2 µm spacing	-

3a-3g, SV2	Living mouse spleen 37 °C	NK1.1 (NK cell), F4/80 (macrophag e)	245.76 ms (245.76 ms) 1 pts	561: 1.0 640: 2.8	-	63×/1.4NA Oil	sLFM, inverted, 13 × 13, 3 × 3	500 AU
			6 ms (245.76 ms) 82 pts	561: 1.0 640: 2.8	1/30		cLFM, inverted, 13 × 13, 1 × 1	11 AU
			6 ms (245.76 ms) 82 pts	561: 1.0 640: 2.8	1/30		csLFM, inverted, 13 × 13, 3 × 3	11 AU
3h-3l, SV3	Living mouse spleen 37 °C	CD11c (dendritic cell), CD8 (T cell)	6 ms (245.76 ms) 493 pts	561: 1.0 640: 1.5	1/30		csLFM, inverted, 13 × 13, 3 × 3	11 AU
4a-4c, SV4	Living mouse liver 37 °C	Ly6G (neutrophil), WGA (vessels)	245.76 ms (245.76 ms) 1 pts	561: 0.2 640: 0.4	-	63×/1.4NA Oil	sLFM, inverted, 13 × 13, 3 × 3	500 AU
			6 ms (245.76 ms) 200 pts	561: 0.2 640: 0.4	1/30		cLFM, inverted, 13 × 13, 1 × 1	11 AU
			6 ms (245.76 ms) 200 pts	561: 0.2 640: 0.4	1/30		csLFM, inverted, 13 × 13, 3 × 3	11 AU
4d-4e	Living mouse liver 37 °C	Ly6G (neutrophil), WGA (vessels)	6 ms (245.76 ms) 106 pts	561: 0.2 640: 0.4	1/30	63×/1.4NA Oil	cLFM, inverted, 13 × 13, 1 × 1	11 AU
			6 ms (245.76 ms) 106 pts	561: 0.2 640: 0.4	1/30		csLFM, inverted, 13 × 13, 3 × 3	11 AU
5a-5c, S19, SV5	Living mouse brain	GCamp6f	44.8 ms (44.8 ms) 12000 pts	488: 0.59	20	25×/1.05NA Water	sLFM, upright, 21 × 21, 3 × 3	1,000 AU
			1.4 ms (44.8 ms) 12000 pts	488: 0.59	20		cLFM, upright, 21 × 21, 1 × 1	26 AU

			1.4 ms<br>(44.8 ms)<br>12000 pts	488: 0.59	20		upright,<br>inverted,<br>21 × 21,<br>3 × 3	26 AU
5d-5i,<br>S20	Living<br>mouse brain	GCamp6f	44.8 ms<br>(44.8 ms)<br>1600 pts	488: 0.59	20	25×/1.05NA<br>Water	sLFM,<br>upright,<br>21 × 21,<br>3 × 3	1,000 AU
			1.4 ms<br>(44.8 ms)<br>1600 pts	488: 0.59	20		csLFM,<br>upright,<br>21 × 21,<br>3 × 3	26 AU
			-<br>(50 ms)<br>1600 pts	920: 100	20		Two-photon<br>microscopy,<br>upright,<br>10 *z*-steps,<br>0.2 μm<br>spacing	-
6a-6d,<br>SV6	Living<br>zebrafish<br>brain	GCamp6s	44.8 ms<br>(44.8 ms)<br>19980 pts	488: 1.18	20	25×/1.05NA<br>Water	sLFM,<br>upright,<br>21 × 21,<br>3 × 3	1,000 AU
			1.4 ms<br>(44.8 ms)<br>19980 pts	488: 1.18	20		cLFM,<br>upright,<br>21 × 21,<br>1 × 1	26 AU
			1.4 ms<br>(44.8 ms)<br>19980 pts	488: 1.18	20		csLFM,<br>upright,<br>21 × 21,<br>3 × 3	26 AU
6e-6g	Living<br>*Drosophila*<br>brain	jGCamp7f	14.1 ms<br>(14.1 ms)<br>25000 pts	488: 0.59	60	25×/1.05NA<br>Water	sLFM,<br>upright,<br>21 × 21,<br>3 × 3	1,000 AU
			0.7 ms<br>(14.1 ms)<br>25000 pts	488: 0.59	60		cLFM,<br>upright,<br>21 × 21,<br>1 × 1	26 AU
			0.7 ms<br>(14.1 ms)<br>25000 pts	488: 0.59	60		csLFM,<br>upright,<br>21 × 21,<br>3 × 3	26 AU
6h-6k,<br>SV7		*pAce*<br>voltage	5.65 ms<br>(5.65 ms)	488: 1.82	150	25×/1.05NA<br>Water	sLFM,<br>upright,	1,000 AU

	Living<br>*Drosophila*<br>brain	indicator	5250 pts				21 × 21,<br>3 × 3<br>virtually	
			0.7 ms<br>(5.65 ms)<br>5250 pts	488: 1.82	150		csLFM,<br>upright,<br>21 × 21,<br>3 × 3<br>virtually	26 AU
S9	Living<br>mouse liver	Ly6G	6 ms<br>(245.76 ms)<br>100 pts	561: 0.2<br>640: 0.4	1/30	63×/1.4NA<br>Oil	csLFM,<br>inverted,<br>21 × 21,<br>3 × 3	11 AU
S11	Living<br>mouse brain	AF647 dye	44.8 ms<br>(44.8 ms)<br>1 pts	640: 0.99	-	25×/1.05NA<br>Water	sLFM,<br>upright,<br>21 × 21,<br>3 × 3,<br>19 *z*-steps,<br>10 µm<br>spacing	1,000 AU
			1.4 ms<br>(44.8 ms)<br>1 pts	640: 0.99	-		csLFM,<br>upright,<br>21 × 21,<br>3 × 3,<br>19 *z*-steps,<br>10 µm<br>spacing	26 AU
S14	300-µm-<br>thick brain<br>slice<br>27 °C	Thy1-YFP	2.8 ms<br>(806.4 ms)<br>1 pts	488: 2.7	-	25×/1.05NA<br>Water	csLFM,<br>upright,<br>21 × 21,<br>3 × 3	26 AU
			-<br>(17 s)<br>1 pts	488: 2.7	-	40×/1.1NA<br>Water	SDCM,<br>inverted,<br>240 *z*-steps,<br>0.25 µm<br>spacing	-
			-<br>(88 s)<br>1 pts	920: 23.4	-	25×/1.05NA<br>Water	Two-photon<br>microscopy,<br>upright,<br>120 *z*-steps,<br>0.5 µm<br>spacing	-
S16	Fixed L929<br>cells<br>27 °C	TSPAN4-<br>mCherry	245.76 ms<br>(245.76 ms)<br>3 pts	561: 1.3	-	63×/1.4NA<br>Oil	sLFM,<br>inverted,<br>13 × 13,	500 AU

							3 × 3	
			6 ms<br>(245.76 ms)<br>3 pts	561: 1.3			csLFM,<br>inverted,<br>13 × 13,<br>3 × 3	11 AU
S17	TBI mice,<br>25 °C	Ly6G<br>(neutrophil),<br>CX3CR1<br>(microglia)	286 pts	920: 37.0	0.115	25×/1.05NA<br>Water	2pSAM,<br>upright,<br>13 angles	-
S21	300-μm-<br>thick brain<br>slice<br>27 °C	Thy1-YFP	6 ms<br>(245.76 ms)<br>1 pts	488: 1.1	-	63×/1.4NA<br>Oil	csLFM,<br>inverted,<br>13 × 13,<br>3 × 3,<br>11 *z*-steps,<br>10 μm<br>spacing	11 AU
