# Supplementary material for: Long-term intravital subcellular imaging with confocal scanning light-field microscopy
Source: Nat Biotechnol. 2024 May 27;43(4):569–80. doi: 10.1038/s41587-024-02249-5 (PMC11994454; doi:10.1038/s41587-024-02249-5)
Supplement: Supplementary file 2 — Reporting Summary [file 41587_2024_2249_MOESM2_ESM.pdf]

nature portfolio

Corresponding author(s): Li Yu, Jiamin Wu, Qionghai Dai

Last updated by author(s): Apr 5, 2024

Reporting Summary

Nature Portfolio wishes to improve the reproducibility of the work that we publish. This form provides structure for consistency and transparency in reporting. For further information on Nature Portfolio policies, see our Editorial Policies and the Editorial Policy Checklist.

Statistics

For all statistical analyses, confirm that the following items are present in the figure legend, table legend, main text, or Methods section.

n/a	Confirmed	
☐	☒	The exact sample size (*n*) for each experimental group/condition, given as a discrete number and unit of measurement
☐	☒	A statement on whether measurements were taken from distinct samples or whether the same sample was measured repeatedly
☐	☒	The statistical test(s) used AND whether they are one- or two-sided<br>*Only common tests should be described solely by name; describe more complex techniques in the Methods section.*
☒	☐	A description of all covariates tested
☒	☐	A description of any assumptions or corrections, such as tests of normality and adjustment for multiple comparisons
☐	☒	A full description of the statistical parameters including central tendency (e.g. means) or other basic estimates (e.g. regression coefficient) AND variation (e.g. standard deviation) or associated estimates of uncertainty (e.g. confidence intervals)
☐	☒	For null hypothesis testing, the test statistic (e.g. *F*, *t*, *r*) with confidence intervals, effect sizes, degrees of freedom and *P* value noted<br>*Give P values as exact values whenever suitable.*
☒	☐	For Bayesian analysis, information on the choice of priors and Markov chain Monte Carlo settings
☒	☐	For hierarchical and complex designs, identification of the appropriate level for tests and full reporting of outcomes
☒	☐	Estimates of effect sizes (e.g. Cohen's *d*, Pearson's *r*), indicating how they were calculated

Our web collection on statistics for biologists contains articles on many of the points above.

Software and code

Policy information about availability of computer code

Data collection	cLFM, sLFM and csLFM imaging data were acquired using LABVIEW (2019 version) and our customized acquisition software. The bead data were acquired using MicroManager software (version 1.4.21). All relevant codes for csLFM are available on Zenodo (https://doi.org/10.5281/zenodo.8198063) and GitHub (https://github.com/THU-IBCS/csLFM-master).
Data analysis	All data processing and analysis were accomplished with customized MATLAB (MathWorks, MATLAB 2019a, R2021a) scripts. The hardware synchronization was controlled with a NI-USB-6363 box and our customized LABVIEW program. The 3D rendering of the volumes was performed by Imaris (Imaris 9.0.1 software) or Voltex modules in Amira (Thermo Fisher Scientific, Amira 2019). The 3D tracking of immune cells in the spleen was carried out automatically using Imaris. The detection of fibers generated from immune cells was carried out using the ridge detection plug-in of the ImageJ software (version 1.51) automatically. Fiber structures with the width of 0.2 to 1 μm and the length of >3 μm were identified, and invalid results were also sifted out manually (Fig. 3h).

For manuscripts utilizing custom algorithms or software that are central to the research but not yet described in published literature, software must be made available to editors and reviewers. We strongly encourage code deposition in a community repository (e.g. GitHub). See the Nature Portfolio guidelines for submitting code & software for further information.

Data

Policy information about availability of data

All manuscripts must include a data availability statement. This statement should provide the following information, where applicable:

- Accession codes, unique identifiers, or web links for publicly available datasets
- A description of any restrictions on data availability
- For clinical datasets or third party data, please ensure that the statement adheres to our policy

Data used for comparisons between sLFM and csLFM has been made publicly available on Zenodo (https://doi.org/10.5281/zenodo.8198063) and GitHub (https://github.com/THU-IBCS/csLFM-master).

Human research participants

Policy information about studies involving human research participants and Sex and Gender in Research.

Reporting on sex and gender	No human research participants in this study
Population characteristics	Not involved in this study
Recruitment	Not involved in this study
Ethics oversight	Not involved in this study

Note that full information on the approval of the study protocol must also be provided in the manuscript.

Field-specific reporting

Please select the one below that is the best fit for your research. If you are not sure, read the appropriate sections before making your selection.

☒ Life sciences ☐ Behavioural & social sciences ☐ Ecological, evolutionary & environmental sciences

For a reference copy of the document with all sections, see nature.com/documents/nr-reporting-summary-flat.pdf

Life sciences study design

All studies must disclose on these points even when the disclosure is negative.

Sample size	The sample size (n) of each experiment is provided in the figure/table legends in the main manuscript and supplementary information files. To ensure statistical significance, sample sizes (n) were all set to n > 3. n varied across experiments, depending on the number of n that can actually be detected.
Data exclusions	No data were excluded for the analysis.
Replication	Biological data shown in Figs. 1-6 and Supplementary Figs. 9, 11-14, 19-20 are representative of n = 6 experiments. Biological data shown in Supplementary Figs. 17, 21 are representative of n = 4 experiments. Characterization data shown in Fig. 2 and Supplementary Figs. 10, 15-16 are representative of n = 6 experiments. Simulated data shown in Supplementary Figs. 2-8, 18 are representative of n = 12 experiments.
Randomization	Randomization was not relevant to this study, since no experimental group was formed.
Blinding	Blinding was not relevant to this study, since no group allocation was performed.

Reporting for specific materials, systems and methods

We require information from authors about some types of materials, experimental systems and methods used in many studies. Here, indicate whether each material, system or method listed is relevant to your study. If you are not sure if a list item applies to your research, read the appropriate section before selecting a response.

Materials & experimental systems

n/a	Involved in the study
☐	☒ Antibodies
☒	☐ Eukaryotic cell lines
☒	☐ Palaeontology and archaeology
☐	☒ Animals and other organisms
☒	☐ Clinical data
☒	☐ Dual use research of concern

Methods

n/a	Involved in the study
☒	☐ ChIP-seq
☒	☐ Flow cytometry
☒	☐ MRI-based neuroimaging

Antibodies

Antibodies used	Ly6G (127607, Biolegend, clone 1A8), CD11c (117307, Biolegend, clone N418), F4/80 (157313, Biolegend, clone QA17A29), CD3 (100235, Biolegend, clone 17A2), NK1.1 (108707, Biolegend, clone PK136)
Validation	All monoclonal antibodies were validated from the website (https://www.biolegend.com, cats# 127607, 164503, 117307, 123107, 100235, 108707), and purchased to perform mouse experiments. According to the description on the website, the application for all of these antibodies is flow cytometric analysis of antibody surface-stained cells. The application references of the five antibodies are Fleming TJ, et al. 1993. J. Immunol. 151:2399. , Lee T, et al. 2014. Mol Biol Cell. 25:583., Schaller E, et al. 2002. Mol. Cell. Biol. 22:8035., Koyama M, et al. 2015. J Exp Med. 212: 1303 - 1321., Carlyle JR, et al. 1999. J. Immunol. 162:5917.

Animals and other research organisms

Policy information about studies involving animals; ARRIVE guidelines recommended for reporting animal research, and Sex and Gender in Research

Laboratory animals	mice (C57BL6/J, Ai148D, ~7-8 weeks), mice (Jax 008451, CX3CR1-GFP, ~8-12 weeks), zebrafish (Tg(huc:GCaMP6s), ~4 days), Drosophila (TH-gal4>UAS-pAce, nsyb-gal4×UAS-GCaMP7f, ~3-8 days).
Wild animals	Not involved in this study.
Reporting on sex	The mice used in this project are male. The biological sex of zebrafish used in the study is unknown. The Drosophila used in this paper are female.
Field-collected samples	Not involved in this study.
Ethics oversight	Animal protocol procedures were reviewed and approved by the Institutional Animal Care and Use Committee office of Tsinghua University.

Note that full information on the approval of the study protocol must also be provided in the manuscript.
